# Radiosynthesis of [^18^F]-Labelled Pro-Nucleotides (ProTides)

**DOI:** 10.3390/molecules25030704

**Published:** 2020-02-06

**Authors:** Alessandra Cavaliere, Katrin C. Probst, Stephen J. Paisey, Christopher Marshall, Abdul K. H. Dheere, Franklin Aigbirhio, Christopher McGuigan, Andrew D. Westwell

**Affiliations:** 1 School of Pharmacy & Pharmaceutical Sciences, Cardiff University, Redwood Building, King Edward VII Avenue, Cardiff CF10 3NB, Wales, UK; alessandra.cavaliere@yale.edu (A.C.);; 2 Wales Research & Diagnostic Positron Emission Tomography Imaging Centre (PETIC), School of Medicine, Cardiff University, University Hospital of Wales, Heath Park, Cardiff CF14 4XN, Wales, UK; katrin.probst@gmx.de (K.C.P.); PaiseySJ@cardiff.ac.uk (S.J.P.); MarshallC3@cardiff.ac.uk (C.M.); 3 Wolfson Brain Imaging Centre and Department of Clinical Neurosciences, University of Cambridge, Cambridge CB2 0QQ, UK; akhd@kcl.ac.uk (A.K.H.D.); fia20@wbic.cam.ac.uk (F.A.)

**Keywords:** fluorination, ProTides, fluorine-18, radiolabelling, PET imaging

## Abstract

Phosphoramidate pro-nucleotides (ProTides) have revolutionized the field of anti-viral and anti-cancer nucleoside therapy, overcoming the major limitations of nucleoside therapies and achieving clinical and commercial success. Despite the translation of ProTide technology into the clinic, there remain unresolved in vivo pharmacokinetic and pharmacodynamic questions. Positron Emission Tomography (PET) imaging using [^18^F]-labelled model ProTides could directly address key mechanistic questions and predict response to ProTide therapy. Here we report the first radiochemical synthesis of [^18^F]ProTides as novel probes for PET imaging. As a proof of concept, two chemically distinct radiolabelled ProTides have been synthesized as models of 3′- and 2′-fluorinated ProTides following different radiosynthetic approaches. The 3′-[^18^F]FLT ProTide was obtained via a late stage [^18^F]fluorination in radiochemical yields (RCY) of 15–30% (*n* = 5, decay-corrected from end of bombardment (EoB)), with high radiochemical purities (97%) and molar activities of 56 GBq/μmol (total synthesis time of 130 min.). The 2′-[^18^F]FIAU ProTide was obtained via an early stage [^18^F]fluorination approach with an RCY of 1–5% (*n* = 7, decay-corrected from EoB), with high radiochemical purities (98%) and molar activities of 53 GBq/*μ*mol (total synthesis time of 240 min).

## 1. Introduction

Clinically approved nucleoside analogues occupy a unique place in drug therapy due to their ability to interfere in biosynthetic and metabolic pathways fundamental to aberrant cellular replication and growth. This is particularly apparent in conditions such as cancer [1] and viral infections [2], where nucleoside analogues are able to inhibit essential human or viral enzymes such as thymidylate synthase or ribonucleotide reductase.

Despite their well-established value in drug therapy, nucleosides suffer from a number of drawbacks as therapeutic agents. Cellular entry of nucleosides through the outer cell membrane requires the active participation of concentrative and equilibrative nucleoside transporters; down-regulation of transporters in cancer cells for example constitutes a known drug resistance mechanism [3]. Following cellular uptake, nucleosides require activation via (normally) three successive enzyme-mediated phosphorylation steps (Figure S1) [4]. The first kinase-mediated phosphorylation is most frequently the rate-limiting step prior to incorporation of the active therapeutic nucleotide tri-phosphate within targets such as DNA/RNA. The requirements for transporter-mediated cellular entry and subsequent phosphorylation to the active form limit the therapeutic efficacy of nucleoside analogues. For example, the anticancer drug gemcitabine, widely used in pancreatic, non-small cell lung, ovarian and breast cancer therapy, is associated with levels of patient response as low as 20% [5].

Given the well-established drawbacks associated with nucleoside analogues, a great deal of research effort has been devoted towards the development of pro-nucleotides [6]. These nucleotide derivatives are defined as analogues able to by-pass the requirement for nucleoside membrane transporters and deliver a masked monophosphate that subsequently breaks down to a nucleotide monophosphate, or nucleotide derivative, within the cell. The most successful approach to this problem has been the phosphoramidate pro-nucleotides (ProTides) pioneered by McGuigan and colleagues at Cardiff University, U.K. [6]. The ProTide approach has revolutionized the field, delivering greatly enhanced concentrations of active nucleotide triphosphate within the diseased cell and improving clinical efficacy in important areas of anticancer and antiviral therapy. Examples of success in the antiviral field include the hepatitis C drug sofosbuvir (Gilead Sciences, Inc., Foster City, CA, USA), launched in 2014 to radically address the high level of unmet medical need in this common disease and achieving the status of the world’s top-selling drug worldwide following launch [7]. Within the anticancer field, several agents have progressed to clinical evaluation with very promising early clinical data. These include the gemcitabine ProTide NUC-1031 (Acelarin), discovered and developed through a collaboration between Cardiff University and NuCana plc. Acelarin is currently in Phase III clinical evaluation in pancreatic cancer [8].

Despite the emerging clear clinical advantages of the ProTide approach, evidence at the molecular level for the accumulation of ProTides at the in vivo site of action is currently lacking. A potential solution to this problem would be to label ProTides such that they were amenable to non-invasive molecular imaging in vivo. In this regard, Positron Emission Tomography (PET) represents an attractive solution. PET imaging is a sensitive and rapidly emerging molecular imaging technology, widely used in prognostic and diagnostic clinical applications [9]. The basis of PET imaging is the incorporation of a radioactive PET-emitting nuclide into the biomarker or drug molecule of interest. The positron released then annihilates close to the site of emission by electron collision, generating two γ-rays (511 Kev) that are detected by coincidence measurement followed by 3D image reconstruction. This annihilation event locates the site of origin of the target molecule with a high level of sensitivity [10]. The choice of PET isotope is crucial; amongst the many options available ^18^F has emerged as the non-metal nuclide of choice due to its intermediate half-life (110 min), relatively low positron energy and exclusive positron mode of decay [11].

The incorporation of ^18^F into small molecules provides significant challenges for the radiochemistry community. The routinely utilized ^18^F-fluoride (dried and purified from aqueous fluoride solution from the cyclotron nuclear reactor source) is poorly nucleophilic, and electrophilic options derived from fluorine gas are disfavoured [12]. In addition, the half-life of ^18^F (110 min) means that ^18^F-incorporation normally has to occur at a late stage of multi-step syntheses, where purification, QC and formulation for patient administration is necessary within a few hours to generate a significant PET signal. These challenges are not insurmountable, and a range of ^18^F-labelled biomarkers and drugs have been produced and applied in clinical medicine. This is best exemplified by the routine use of the gold standard PET biomarker [^18^F]FDG (fluorodeoxyglucose) in cancer diagnosis, staging and monitoring of response to therapy [13].

Building on our previous experience of ^18^F-radiolabelling of nucleosides for PET imaging [14,15], we set out to apply our technology to the ProTide field. Here we report the first radiosynthetic routes to both 3′- and 2′-fluorinated model ProTides. Application of this technology could help to improve both diagnostic and prognostic applications of ProTide technology in the clinic, with profound implications for this exciting area of anticancer and antiviral therapy.

## 2. Results and Discussion

**Figure 1:**
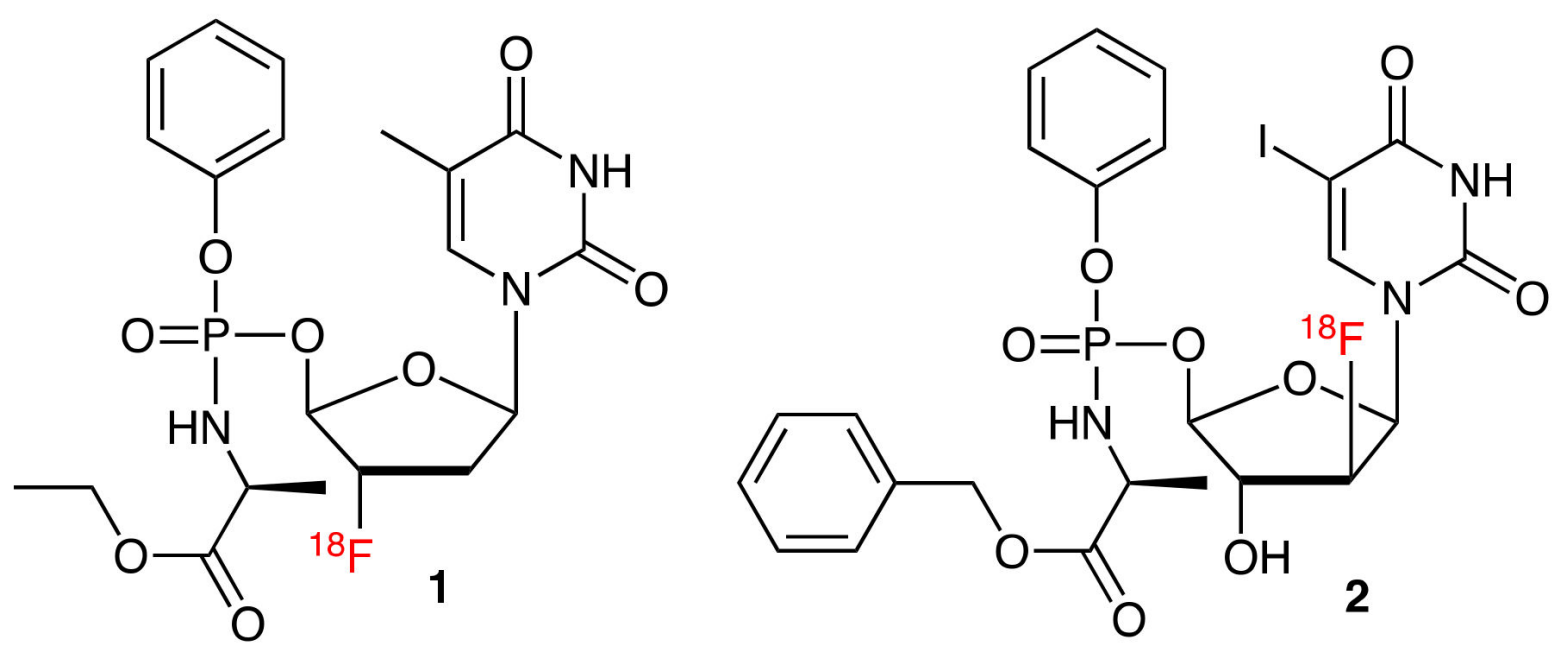
Structures of the [^18^F]FLuoroThymidine (FLT) ProTide (**1**) and [^18^F]Fluoro-Iodo-ArabinofuranosylUracil (FIAU) (ProTide (**2**).

As a proof of concept, two [^18^F]-radiolabelled ProTides have been synthesised. The [^18^F]**FL**uoro**T**hymidine (FLT) ProTide (**1**) and the [^18^F]Fluoro-Iodo-ArabinofuranosylUracil (FIAU) ProTide (**2**) (Figure 1) were our chosen model standards for 3′-fluorinated and 2′-fluorinated ProTides, respectively.

### 2.1. [^18^F]FLT- a Prototypical 3′-fluorinated ProTide

**Scheme 1:**
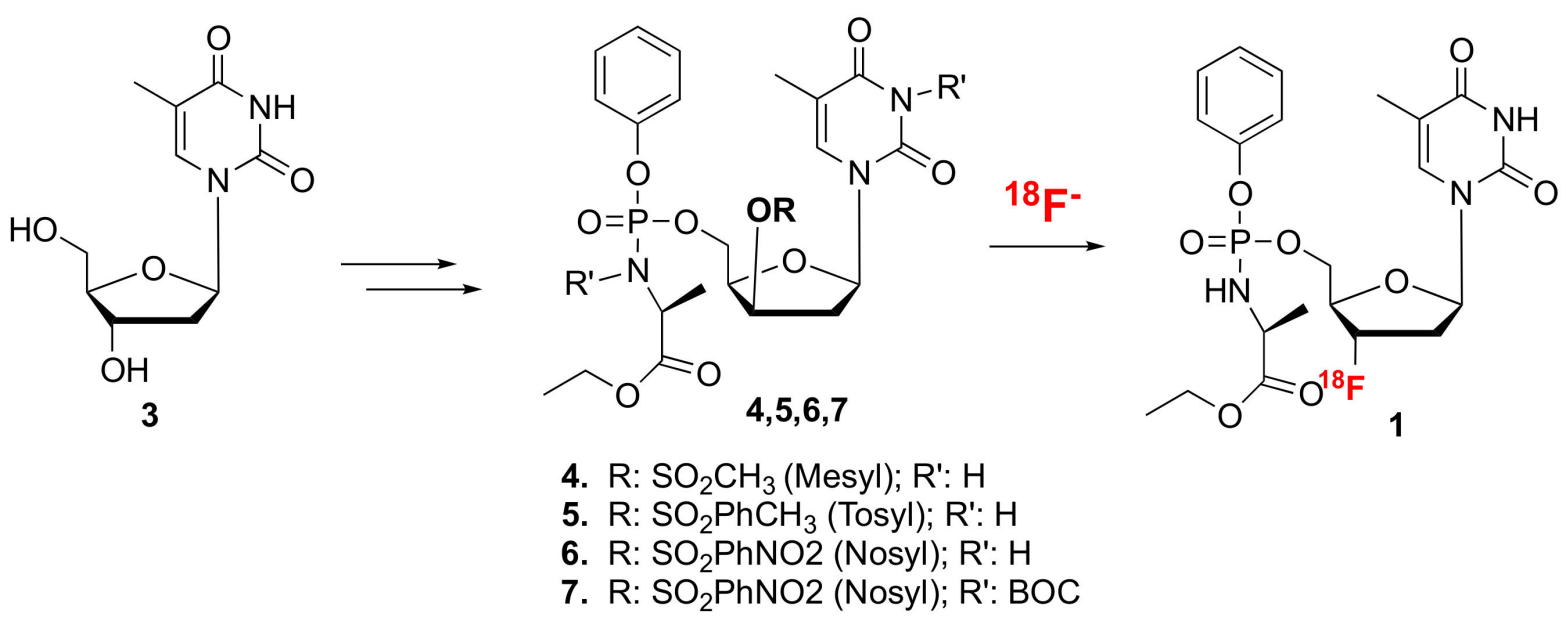
Late stage fluorination approach for the synthesis of the [^18^F]FLT ProTide (**1**).

[^18^F]FLT, a 3′-fluorinated nucleoside, is an established PET imaging agent used as a tumour proliferation biomarker [16]. Synthetic approaches involving a late stage [^18^F]-fluorination of different precursor molecules of [^18^F]FLT have been extensively studied [15]. Moreover, our group has previously reported the synthesis of a series of (non-radiolabelled) FLT ProTides that showed a relatively safe toxicological profile compared to the parent nucleoside as well as moderate anti-HIV activity [17]. For these reasons, [^18^F]FLT ProTide has been selected as a target compound in this study to represent the class of 3′-fluorinated ProTides. The choice of the phenol group as aromatic moiety and the L-alanine ethyl ester as the amino acid ester on the phosphoramidate moiety were dictated by the accessibility of the starting materials as well as the favourable yields associated with the coupling reactions involved in the synthesis [17]. The radiochemical synthesis of [^18^F]FLT ProTide (**1**) was planned accordingly taking into account the short half-life of fluorine-18 (110 min), with the [^18^F]fluorination occurring at a late stage in the synthesis (Scheme 1). The challenge for this radiosynthetic plan was therefore to identify a precursor molecule with a balanced reactivity towards the weakly nucleophilic [^18^F]fluoride with stability in the harsh thermal conditions used for the radiolabelling step. A series of good leaving groups {methanesulfonyl (mesyl); *p*-toluenesulfonyl (tosyl); *p*-nitrophenylsulfonyl (nosyl)} for the key nucleophilic fluoride displacement reaction (intermediates **4**–**7**) were selected for reaction optimization.

#### 2.1.1. Synthesis of the Cold FLT ProTide Standard

**Scheme 2:**
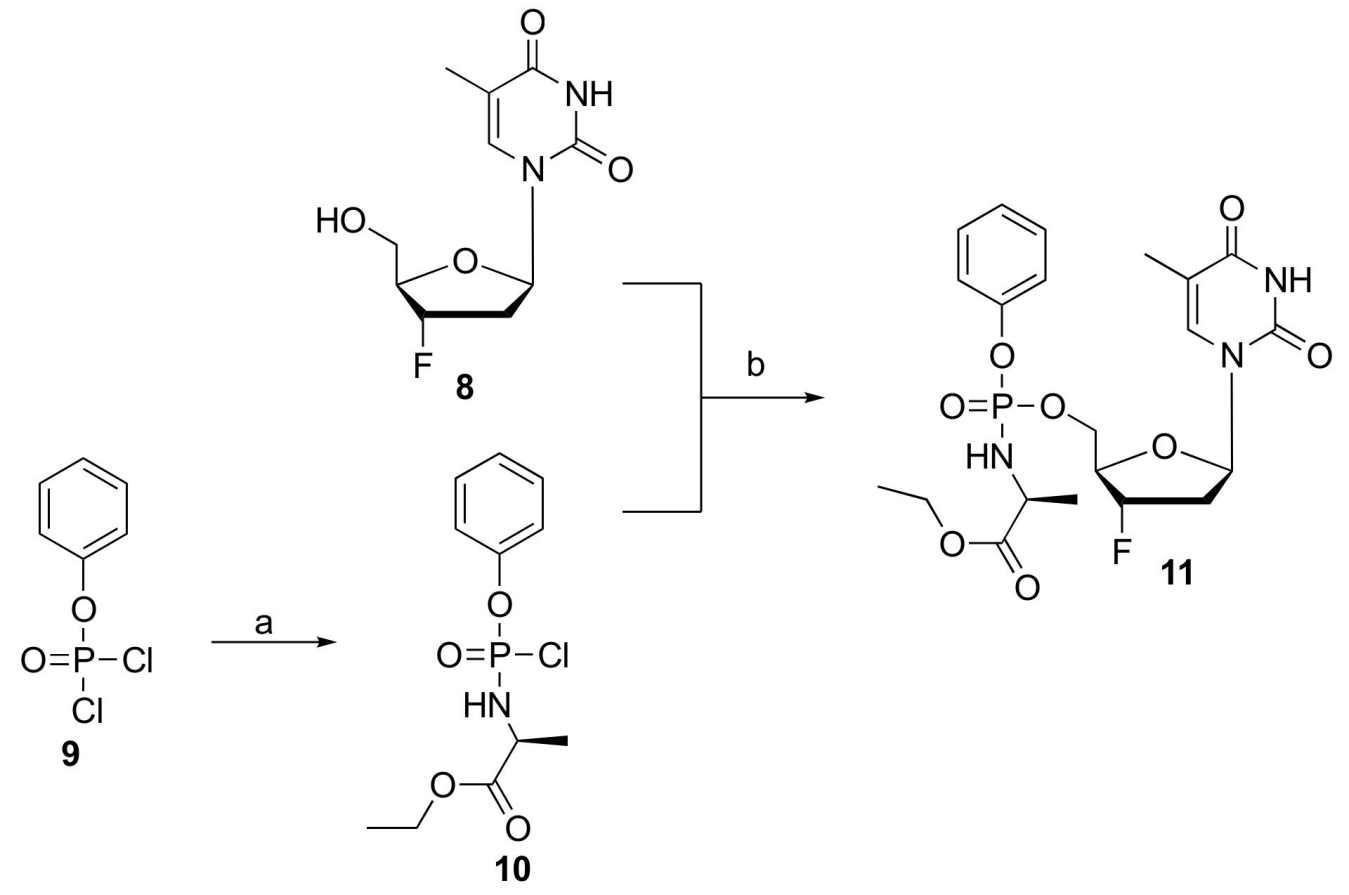
Synthesis of the cold standard FLT ProTide. Reagents and conditions: (a) l-alanine ethyl ester hydrochloride salt, Et_3_N, −78 °C to rt, anh. CH_2_Cl_2_, 3 h, 92%; (b) *t*-BuMgCl, anh. THF, 18 h, 24%.

A cold non-radioactive standard of the [^18^F]FLT ProTide was synthesized according to established ProTide chemistry protocols (Scheme 2) [18]. The phosphorochloridate intermediate (**10**) was first obtained from the L-alanine ethyl ester hydrochloride salt and the commercially available dichlorophosphate (**9**) using triethylamine as base. Compound **10** was obtained as a mixture of diastereoisomers because of the formation of a new stereocenter at the phosphorus atom in a 1:1 Rp: Sp ratio. Commercially available FLT (**8**; Carbosynth) was then reacted with the phosphorylating reagent using *tert*-butyl magnesium chloride (*t*-BuMgCl) as a hindered base. The desired product (**11**) was obtained as a mixture of diastereoisomers (1:1 Rp:Sp) with a yield of 24% and was used as standard analytical control for studies of the radiochemical synthesis of [^18^F]FLT ProTide.

Stability studies were performed on the non-radioactive standard to test the susceptibility of the ProTide phosphoramidate to the high temperatures used during the [^18^F]fluorination. The dynamic behaviour of the phosphoramide moiety was therefore monitored at temperatures ranging from 50 °C to 120 °C. ^31^P NMR spectroscopy is particularly well suited for this purpose considering the characteristic chemical shifts at around δ 4 ppm of the phosphoramidate backbone of the FLT ProTide [17]. The two ^31^P NMR peaks of the diasteroisomeric mixture were observed to be stable when the compound was heated up to 120 °C, confirming its stability to the high temperatures that were used during the radiolabelling step (see Supplementary Materials, Figure S2).

#### 2.1.2. Synthesis of a Series of Organophosphates as Precursor Molecules of the [^18^F]FLT ProTide

To design a late stage fluorination for the class of 3′-substituted ProTides, a multi-step synthesis was performed to obtain a thymidine based ProTide with an anhydroxylic group in the 3′-β position of the ribose ring. The first step consisted of the formation of the 3′-β hydroxy intermediate (**13**) via a Mitzunobu reaction [18] followed by hydrolysis of the intermediate compound **12** to obtain inversion of the stereochemistry of the hydroxylic group at the 3′ position of the thymidine. The intermediate **14** was again synthesised following the standard procedure previously described. *N*-methylimidazole (NMI) was used at this time as the coupling reagent because of the presence of the free hydroxylic group in the 3′-position that could compete with the 5′-OH group for the phosphorylation [19].

**Scheme 3:**
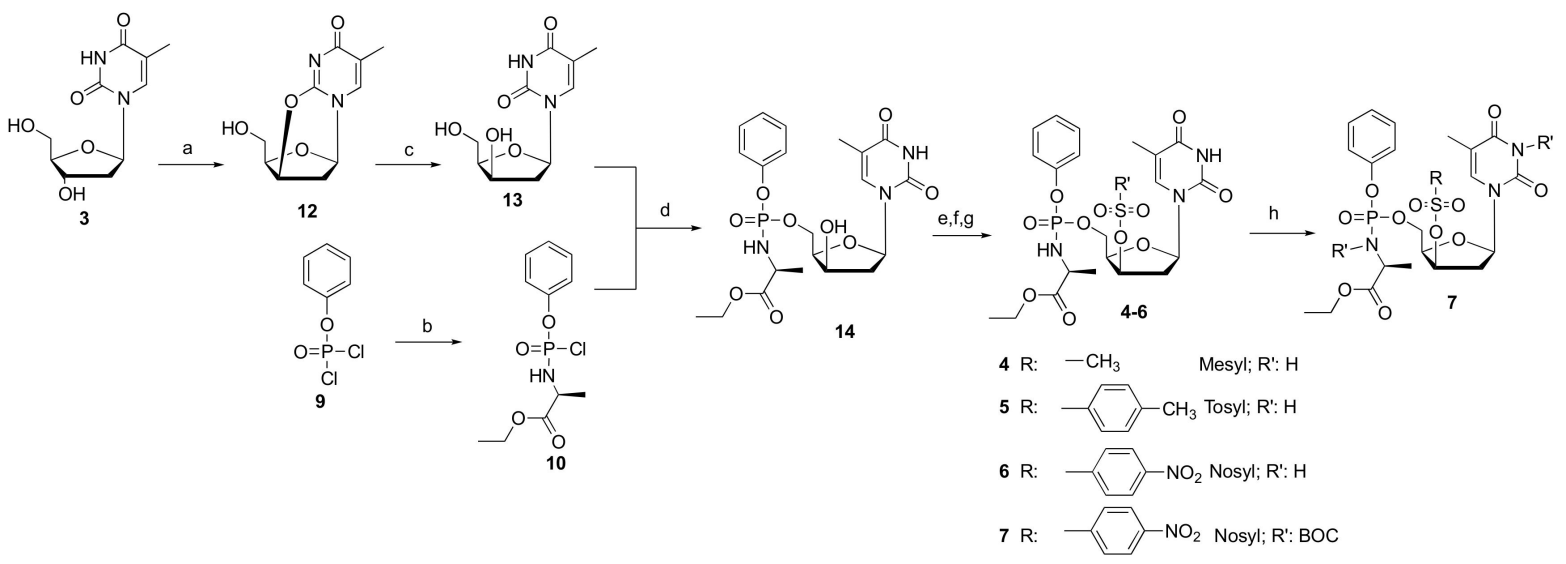
Synthetic procedure for the mesyl, tosyl and nosyl precursors. Reagents and conditions: (a) PPh_3_, DIAD, anh. CH_3_CN, −20 °C to 0 °C, 5 h, 52%; (b) l-alanine ethyl ester hydrochloride salt, Et_3_N, −78 °C to rt, anh. CH_2_Cl_2_, 3 h, 92%; (c) NaOH (1.5 M), CH_3_OH, 90 °C, 3 h, 64%; (d) Phosphorochloridate, NMI, anh. THF, 25 °C, 18 h, nitrogen atm, 18.4%; (e) Mesyl chloride, Et_3_N, anh. CH_2_Cl_2_, nitrogen atm., 0 °C to 25 °C, 1.5 h, 29.5%; (f) Tosyl chloride, pyridine, AgOTf, 0 °C to rt, 2 h, 35%; (g) Nosyl chloride, pyridine, AgOTf, 0 °C to rt, 2 h, 60%; (h) Di-*tert*-butyldicarbonate, pyridine, rt, 16 h, 56%.

The hydroxyl group at the 3′-β position of **14** is a poor leaving group for the nucleophilic substitution reaction with anhydrous [^18^F]fluoride. Therefore, the 3′-hydroxyl group was selectively activated with a series of sulfonic esters to produce good leaving groups (**4**–**6**) for reaction with the weakly nucleophilic [^18^F]fluoride, in accordance with literature precedent [11,20]. The intermediate **14** was reacted with mesyl chloride, tosyl chloride and nosyl chloride respectively in presence of a weak base such as pyridine or Et_3_N with or without AgOTf as a catalyst (Scheme 3). To improve the stability of precursor **6** and avoid competitive cyclization/elimination reaction upon reaction with the fluorine-18 [21], protection of the NH group of the pyrimidine ring was performed with the *tert*-butoxycarbonyl group (Boc). Surprisingly, the major product of the reaction observed was the di-protected ProTide (**7**) bearing Boc groups at both the NH of the pyrimidine ring and the phosphoramidate moiety. The abundance of the di-Boc protected compound compared to the mono-protected product as result of the Boc-protection reaction, together with the need for a stable fluorination precursor, led us to choose the di-Boc protected product (**7**) for the following radio-fluorination step.

#### 2.1.3. Radiochemical Synthesis of the [^18^F]FLT ProTide

The Eckert & Ziegler modular lab was used for the [^18^F]-fluorination following the schematic described in the Supplementary Material (Figure S3). K [^18^F]F/K_222_/K_2_CO_3_ was used as the fluorinating agent and a series of solvents and temperatures were tested to establish the best conditions for the radio-fluorination. The methanesulfonyl (mesyl) precursor (**4**) and the *p*-toluenesulfonyl (tosyl) precursor (**5**) did not give the expected [^18^F]-radiolabelled compound as observed from the radio HPLC chromatograms (Figures S4 and S5 and Tables S1 and S2). The *p*-nitrobenzenesulfonate (nosyl) precursor **6** was, as expected, the most reactive among the three organosulfonate leaving groups for the S_N_2 reaction with the weak nucleophile [^18^F]fluoride [11], but lacked stability with the formation of multiple radiolabeled polar compounds and a radiochemical yield < 1% as determined by analytical HPLC (Figure S6 and Table S3).

**Scheme 4:**
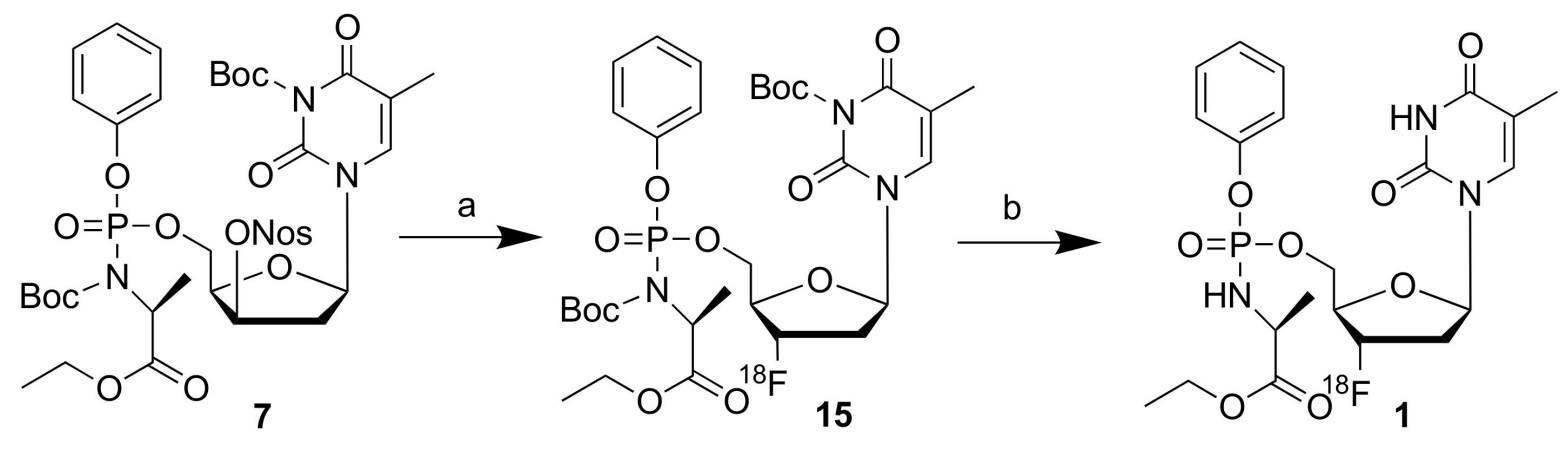
^18^F-fluorination of the Boc protected nosyl derivative **7** and deprotection. Reagents and conditions: (a) ^18^F^−^, Kryptofix, anh. CH_3_CN, 90 °C, 30 min.; (b) 2 N HCl, 95 °C, 10 min.

To increase the stability of the precursor, two Boc protecting groups were added, as previously described, leading to the formation of compound **7**. This precursor proved to be the best substrate for [^18^F]-fluorination. Radio-HPLC showed a major product (**15**) with a retention time at around 15 min (Figure S7). This suggested that the desired Boc radiolabelled product was formed therefore supporting the hypothesis that the Boc double protection provides improved stability for the nosyl precursor. The deprotection step was then carried out by adding 2 N HCl for 10 min at 95 °C [22] and the final compound was then neutralised with a 2 M NaOH solution. Gratifyingly the major product of this reaction was the [^18^F]FLT ProTide (**1**) with few other minor by-products (Scheme 4, Figure S8).

**Figure 2:**
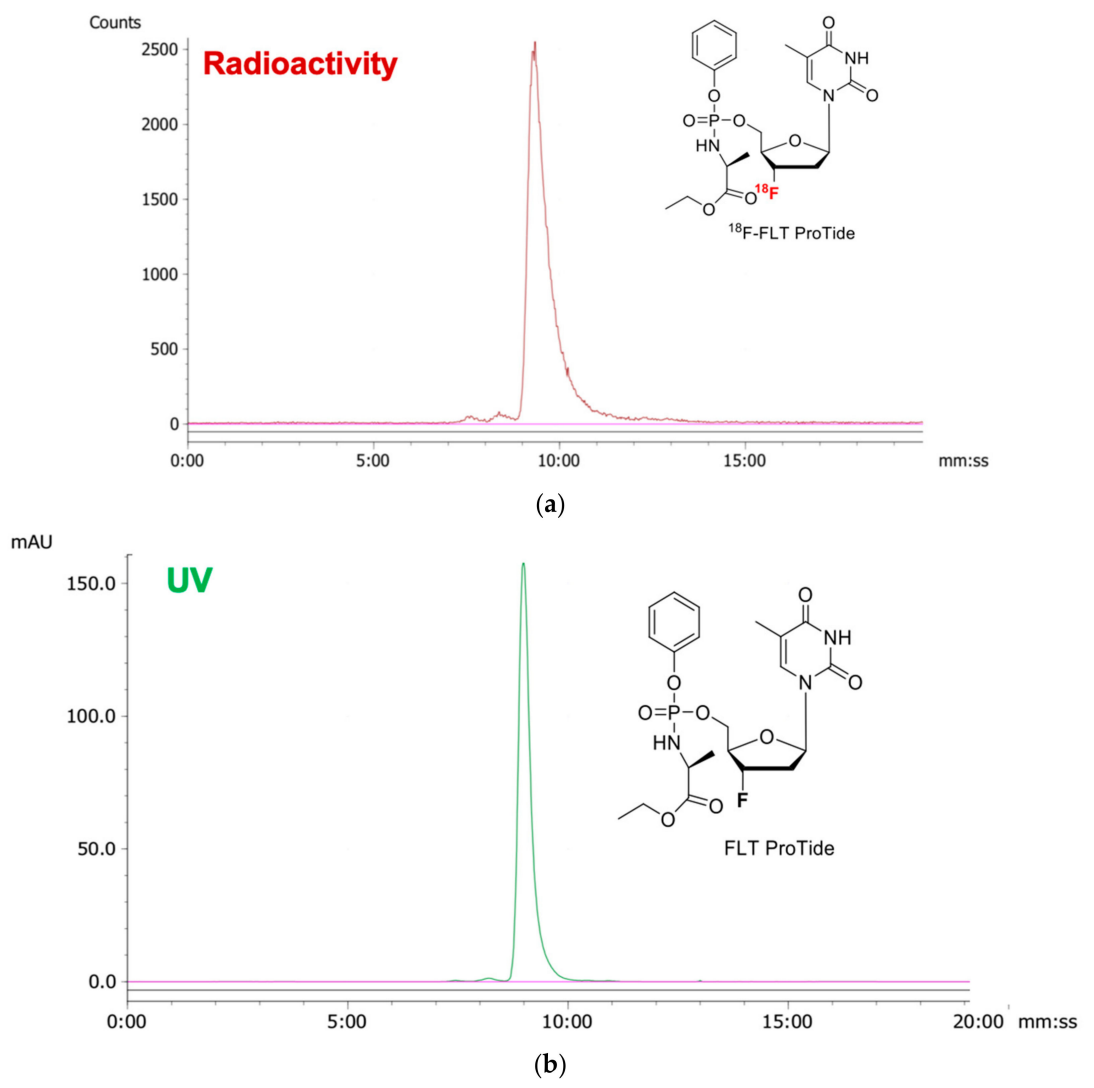
HPLC of the [^18^F]FLT ProTide (**1**). (**a**) Radioactive chromatogram of the purified [^18^F]FLT ProTide with R_t_ of 9.5 min; (**b**) UV chromatogram of the reaction mixture co-spiked with the cold standard (R_t_: 9.2 min). HPLC system: 90% H_2_O/10% CH_3_CN, to 50% H_2_O/50% CH_3_CN.

The compound was then purified by semi-preparative HPLC (Phenomenex Synergi 4μ Hydro-RP 80, C-18, 10 × 250 mm) and was eluted after 35 min at a flow rate of 3.5 mL/min using 30% CH_3_CN/70% H_2_O as the mobile phase. To confirm the identity of the ^18^F-product (**1**), an aliquot of the purified sample was analysed by HPLC (Phenomenex Synergi 4μ Hydro-RP 80, C-18, 4.6 × 250 mm) via co-elution with the cold standard. The ^18^F-product showed a R_t_ of 9.5 min and the cold standard co-injected eluted at a Rt of 9.2 min (Figure 2) confirming the identity of the [^18^F]FLT ProTide (**1**). Radiochemical reactions were carried out using starting activities between 1.5–8 GBq, leading to final product activities of 300–580 MBq in a good radiochemical yield (RCY) of 15–30% (*n* = 5, decay-corrected from end of bombardment (EoB)). High radiochemical purities (≥97%) and molar activities of 56 GBq/μmol were obtained, and the total synthesis time was 130 min after the end of bombardment (EoB).

### 2.2. [^18^F]FIAU - A Prototypical 2′-Fluorinated ProTide

**Scheme 5:**
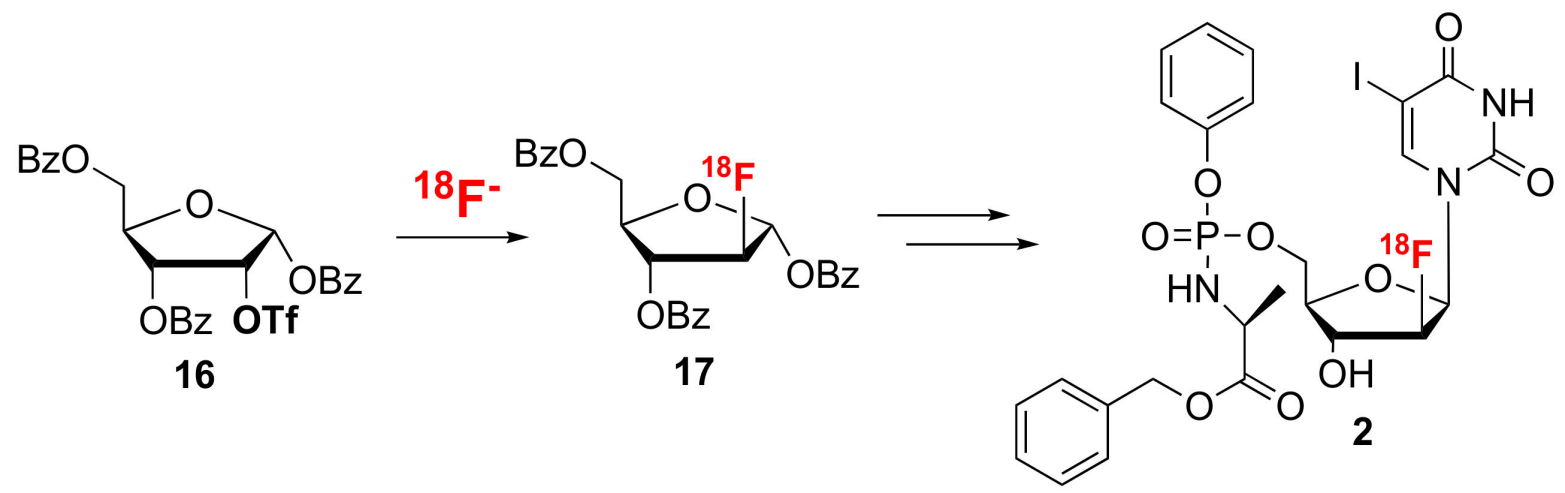
Synthetic approach for the radiosynthesis of the [^18^F]FIAU ProTide (**2**).

2′-deoxy-2′-[^18^F]fluoro-1-β-D-arabinofuranosyl-5-iodouracil ([^18^F]FIAU), is a PET biomarker used for imaging HSV1-*tk* gene expression in biological processes including transcriptional regulation, lymphocyte migration and stem-cell tracking [23]. Building on previous developed radiosyntheses of this tracer for PET imaging, we decided to synthesise a ProTide of [^18^F]FIAU (**2**) as a model of the class of the 2′-fluorinated ProTides [10] introducing ^18^F early in the synthetic sequence as outlined in Scheme 5.

#### 2.2.1. Synthesis of the Non-Radioactive FIAU ProTide Standard 

**Scheme 6:**
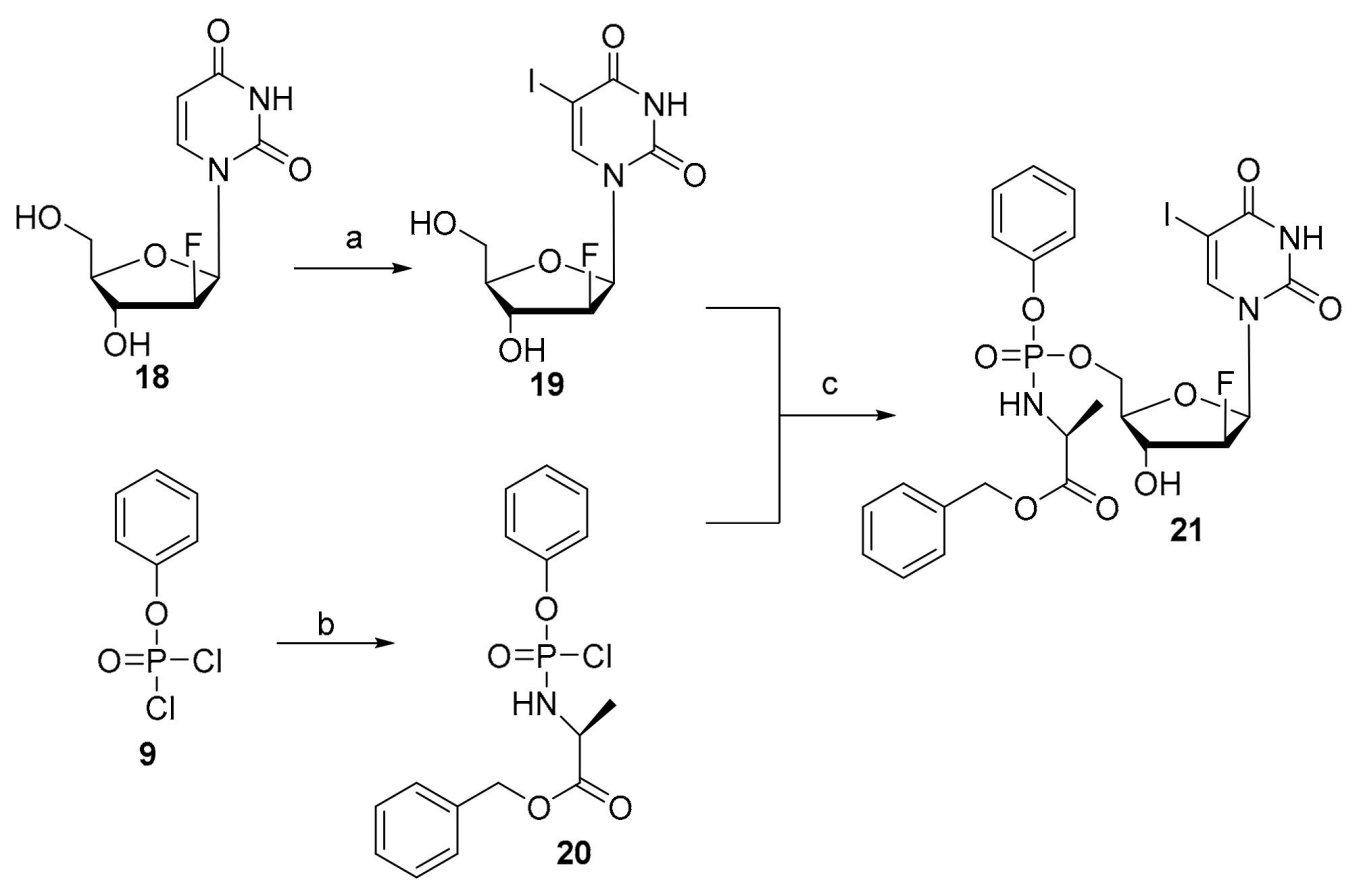
Synthesis of the non-radioactive standard FIAU ProTide (**21**). Reagents and conditions: (a) I_2_, Ceric ammonium nitrate, ACN, 75 °C, 1 h, 60%; (b) l-alanine benzyl ester hydrochloride salt, Et_3_N, −75 °C to rt, anh. CH_2_Cl_2_, 3 h, 88%; (c) NMI, anh. THF, 0 °C to rt, 16 h, 10%.

A cold standard of FIAU ProTide (**21**) was synthesised following the synthesis outlined in Scheme 6. The commercially available compound **18** was firstly iodinated at the C-5 position upon reaction with iodine and cerium ammonium nitrate to give compound **19** under previously reported conditions [24]. The ProTide **21** was synthesised using methodology described above with the exception that the L-alanine ethyl ester was here replaced by a benzyl ester (using compound **20**). The phosphoramidate **21** was obtained as a diasteroisomeric mixture for co-injection with the radiolabelled counterpart, the [^18^F]FIAU ProTide, to confirm its identity by HPLC.

#### 2.2.2. Radiochemical Synthesis of the [^18^F]FIAU ProTide

The first step consisted of the radioactive fluorination of the commercially available sugar **16** bearing a triflate as leaving group according to literature precedent ([^18^F]fluoride, Kryptofix, anh. CH_3_CN, 95 °C) [25]. The reaction was again carried out using the E&Z modular lab. After purification with an alumina sep-pak [26], the radiolabelled sugar (**17**) was used for the next step without further purification. When an aliquot of the radioactive mixture was co-spiked with a cold standard (Sigma Aldrich), it showed the same retention time at around 3 min (Figure S10).

**Scheme 7:**
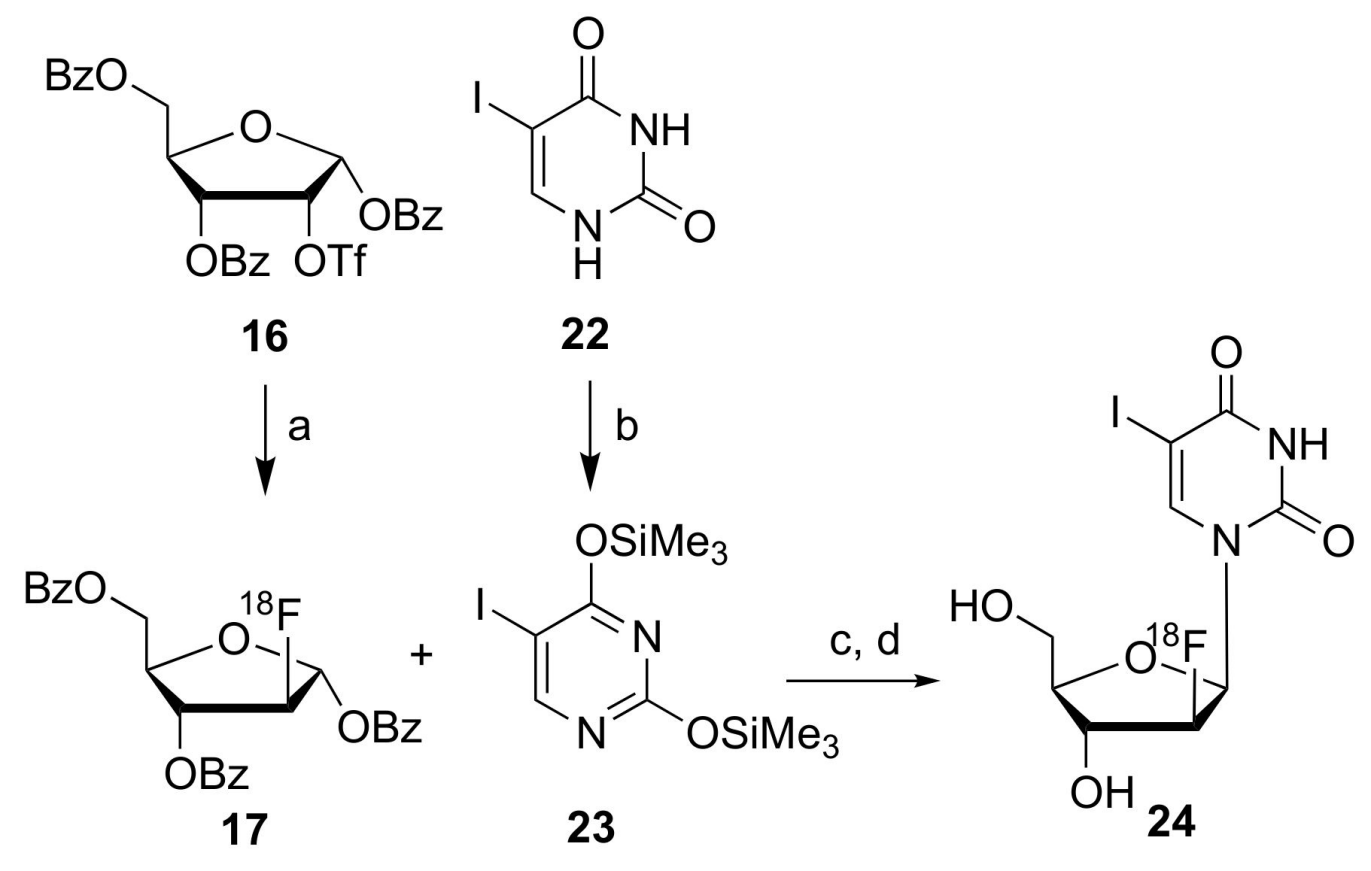
Radiochemical synthesis of [^18^F]FIAU (**24**). Reagents and conditions: (a) ^18^F^-^, Kryptofix, anh. CH_3_CN, 95 °C, 30 min; (b) Hexamethyldisilazane, TMSOTf, anh. dichloroethane, 85 °C, 2 h; (c) anh. CH_3_CN, 85 °C, 1 h; (d) NaOCH_3/_CH_3_OH, 85 °C, 10 min.

The second step consisted in the protection of the base moiety **22** with hexamethyldisilazane and the catalyst trimethylsilyl trifluoromethanesulfonate (TMSOTf) [27] to obtain compound **23** that was coupled with the radiolabelled sugar (**17**) without further purification. The glycosylation reaction led to the formation of two anomers following removal of the TMS groups under basic conditions, the β-anomer ([^18^F]FIAU) (**24**) and the α-anomer in a ratio 2:1 (Figure S10). Attempts to increase the speed of the reaction by either reducing the time or using a combination of catalysts (TMSOTf and SnCl_4_) led to incomplete conversion into the final product or favoured the formation of the α-anomer (ratio β:α = 1:1.3), as shown in Table 1 [27,28]. Therefore, based on these attempts to optimise the reaction conditions, the synthetic pathway in Scheme 7 was established as the most suitable for synthesis of [^18^F]FIAU **24**.

**Figure 3:**
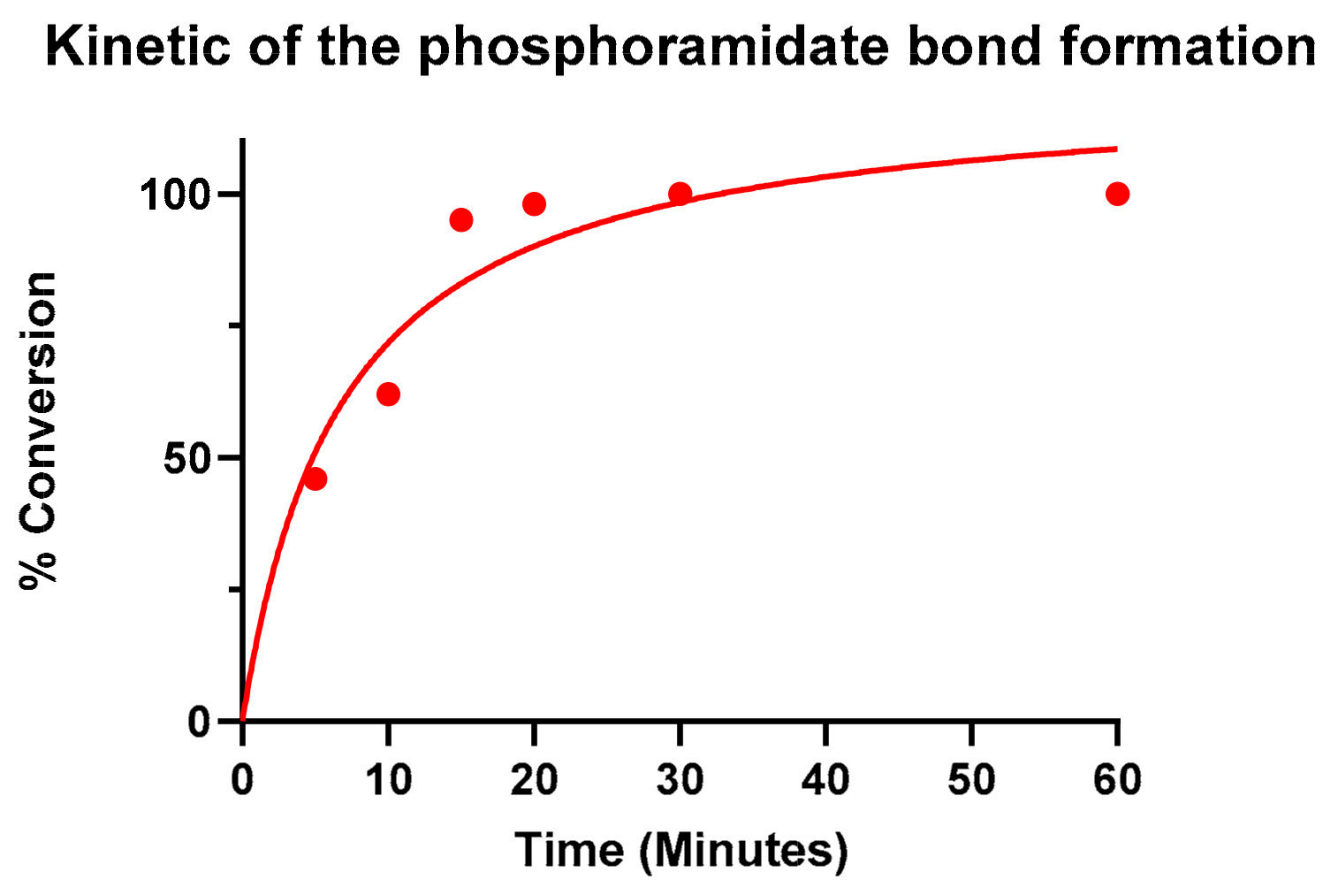
Conversion rate of FIAU (**19**) into FIAU ProTide (**21**): % conversion to FIAU ProTide during the coupling reaction was calculated over time at 50 °C using ^31^P NMR spectroscopy and analytical HPLC chromatography.

Finally the last step consisted of the coupling between the [^18^F]FIAU (**24**) and the appropriate phosphorochloridate previously synthesised according to the standard NMI promoted procedure [19]. The phosphoramidate reaction between phosphorochloridate and nucleoside under non-radioactive conditions is reported in the literature as a room temperature reaction over 16h [19]. However, this procedure would not be suitable for a reaction as time sensitive as one involving the short-lived radionuclide fluorine-18 (t_1/2 =_ 109.7 min.). For this reason, we developed an assay to observe the progress of the phosphoramidate bond formation. The substrate of this assay was the non-radioactive FIAU and the reaction was monitored via ^31^P NMR spectroscopy and HPLC chromatography. Surprisingly we observed almost complete conversion into the ProTide after 15 min when the reaction was conducted at mild temperatures (50 °C) to then reach a steady state at around 30 min (Figure 3).

**Scheme 8:**
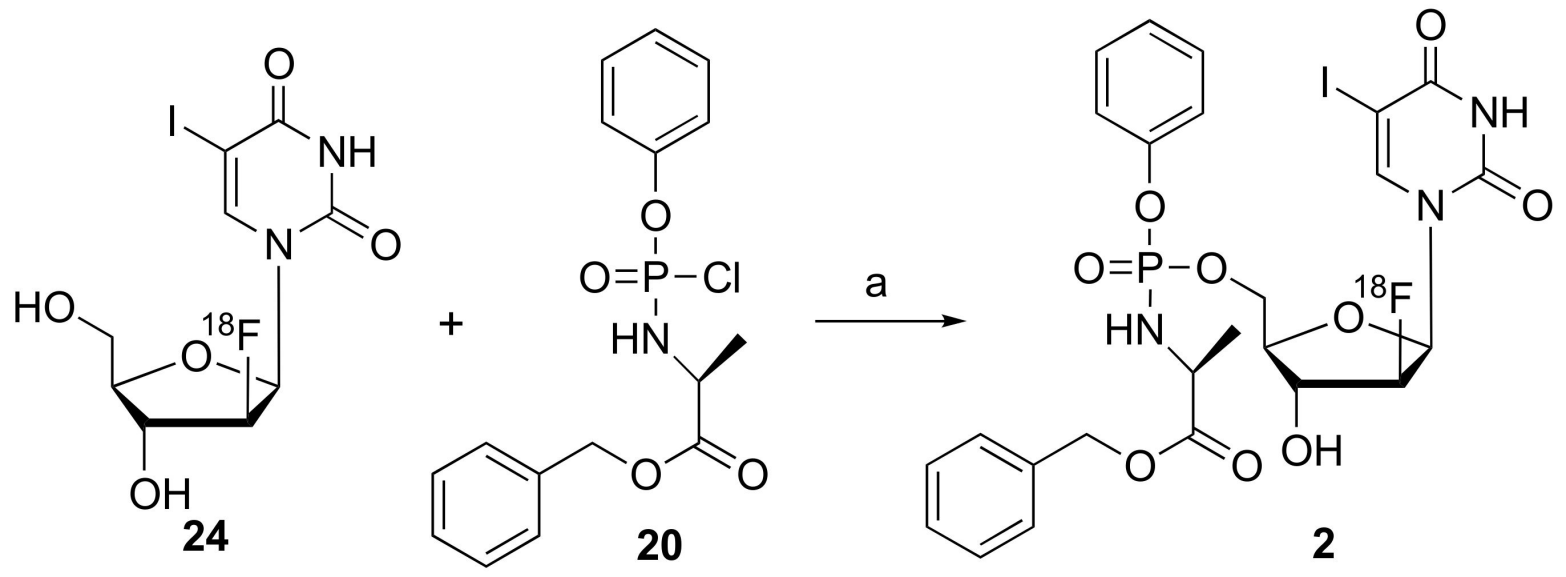
Synthesis of the [^18^F]FIAU ProTide. Reagents and conditions: (a) NMI, anh. THF, 50 °C, 20 min.

We therefore applied the same conditions for the radioactive reaction and observed formation of the final compound (**2**) at 50 °C after 15–20 min. (Scheme 8).

**Figure 4:**
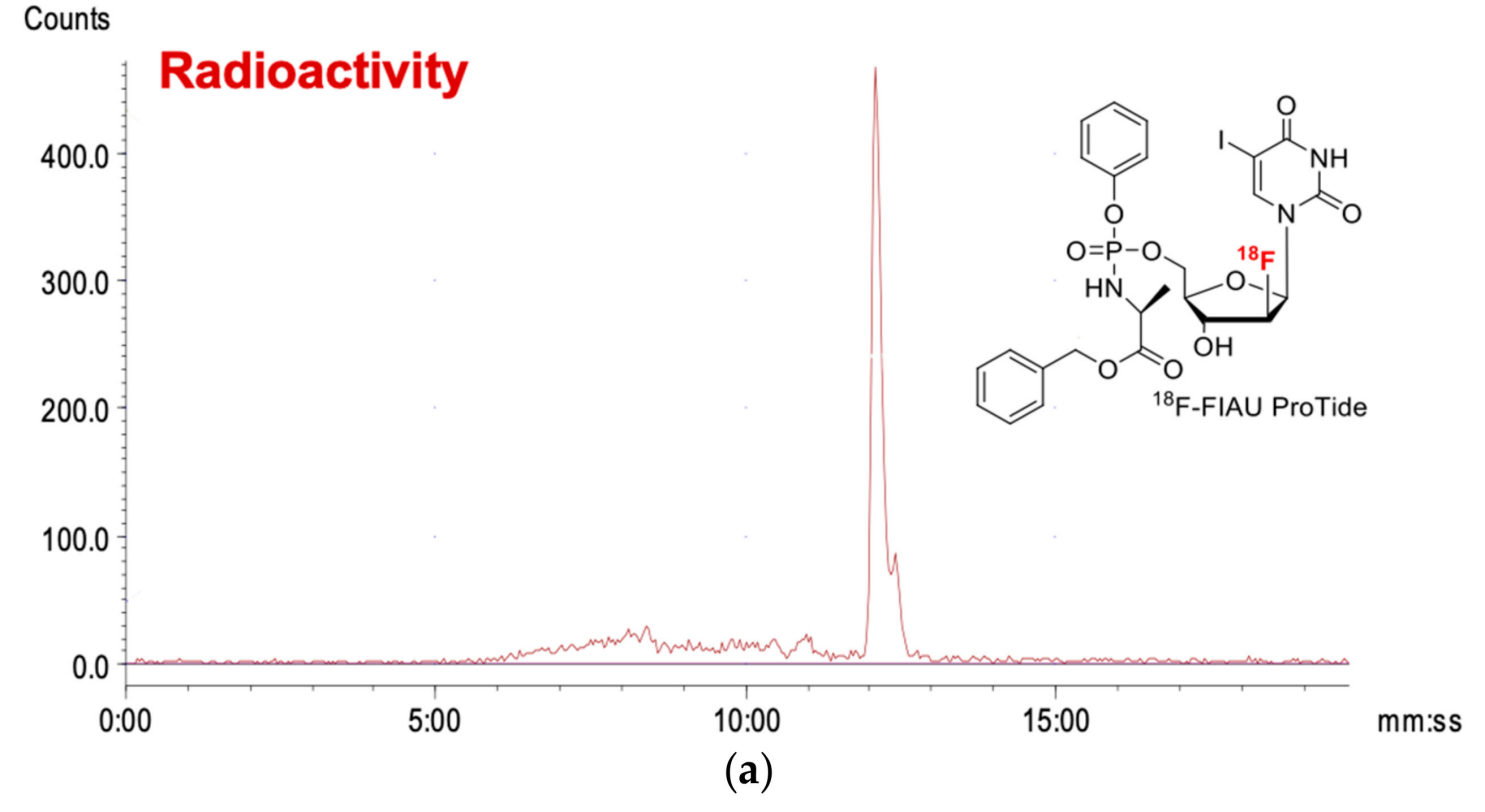

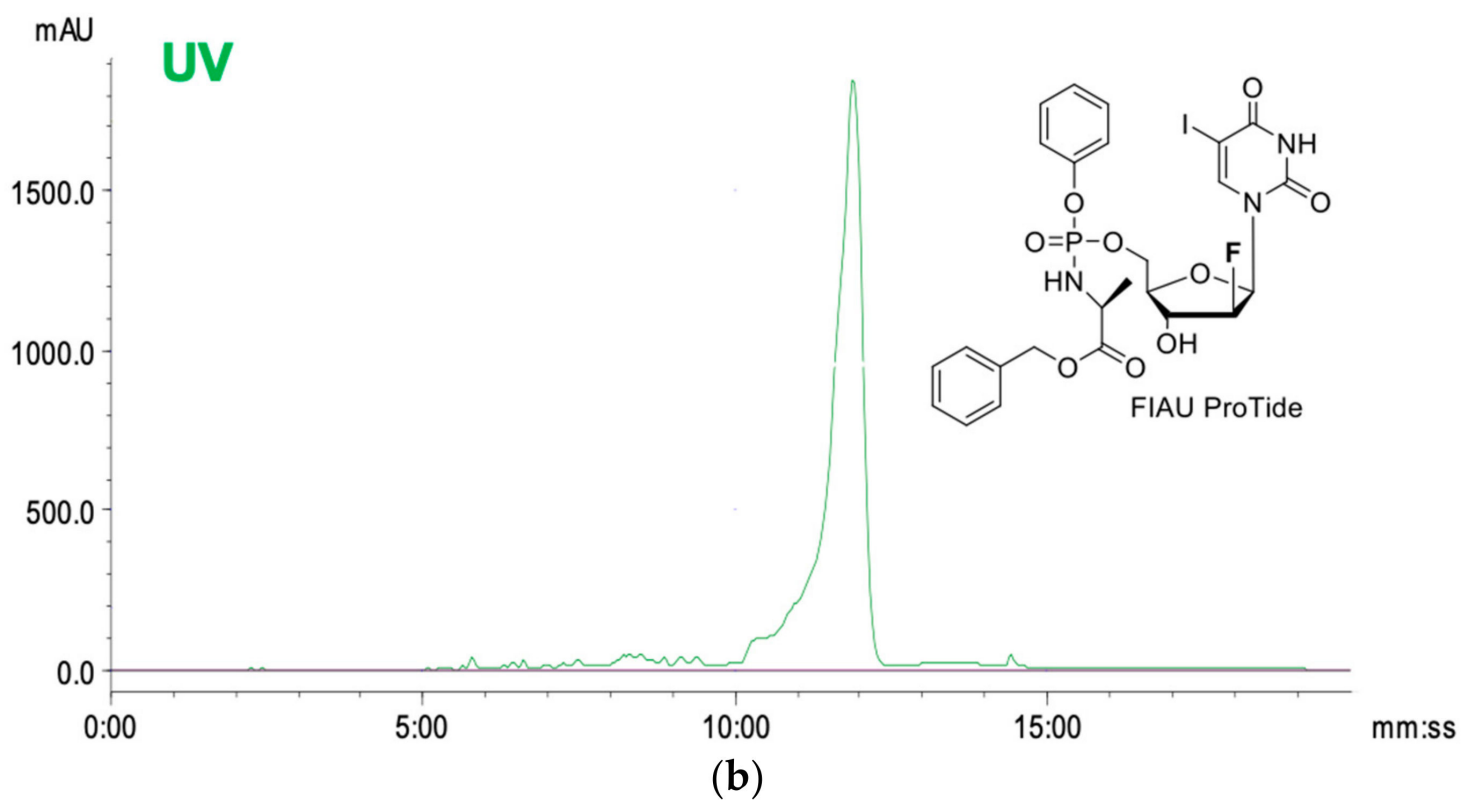
HPLC of [^18^F]FIAU ProTide. (**a**) Radioactive chromatogram of the purified [^18^F]FIAU ProTide with R_t_ of 12.3 min; (**b**) UV chromatogram of the [^18^F]FIAU ProTide co-spiked with the non-radioactive standard. (R_t_ of non-radioactive standard: 12.2 min). HPLC system: 90% H_2_O/10% CH_3_CN to 50% H_2_O/50% CH_3_CN.

Satisfyingly, when an aliquot was taken to perform an analytical HPLC evaluation, [^18^F]FIAU ProTide (**2**) was observed to be the main product of the reaction (Figure S11). The product was then isolated via semi preparative HPLC and was eluted after 23 min at a flow rate of 3.5 mL/min using 50% CH_3_CN/50% H_2_O as the mobile phase. An aliquot of the purified sample was analysed by analytical HPLC via co-elution with the non-radioactive standard (Figure 4).

Radiochemical reactions were carried out using starting activities between 7–15 GBq, leading to final product activities of 8–46 MBq in RCY of 1–5% (*n* = 7, decay-corrected from end of bombardment (EoB)), with high radiochemical purities (98%) and molar activities of 53 GBq/*μ*mol. The total synthesis time was 240 min after the end of bombardment (EoB), within the acceptable range of just over two half-lives for future pre-clinical/clinical applications.

## 3. Materials and Methods

### 3.1. General Non-Radioactive Chemistry: Reagents and Analytical Methods

All the reagents and anhydrous solvents were purchased from Sigma-Aldrich. FLT was purchased from Carbosynth Ltd. (Berkshire, UK). Fluka silica gel (35–70 mm) was used as stationary phase for column chromatography. ^1^H NMR spectra were acquired for all known compounds whereas for novel compounds ^1^H NMR, ^31^P NMR, ^13^C NMR, MS and HPLC data were acquired. ^1^H NMR were measured using a Bruker Advance Ultra Shield spectrometer (500 MHz) at ambient temperature. Data were recorded as follows: chemical shift in δ ppm from internal standard tetramethylsilane; multiplicity (*s* = singlet; *d* = doublet; *t* = triplet; *m* = multiplet); coupling constant (Hz); integration and assignment. ^13^C NMR spectra were measured using a Bruker Advance Ultra Shield spectrometer (125 MHz) at ambient temperature. Chemical shifts were recorded in ppm from the solvent resonance used as the internal standard (e.g., CDCl_3_ at 77.00 ppm). ^31^P NMR spectra were recorded on a Bruker Advance Ultra Shield spectrometer (202 MHz) at ambient temperature. ^19^F NMR spectra were recorded on a Bruker Advance Ultra Shield (474 MHz) spectrometer at ambient temperature. High-performance liquid chromatography (HPLC) analysis was conducted on an Agilent Technology 1200 Series System at the PET imaging centre in Cardiff (PETIC) with an analytical reversed phase column (Phenomenex Synergi 4μ Hydro-RP 80, C-18, 4.6 × 250 mm). Thin-layer chromatography (TLC) was conducted on pre-coated silica gel 60 GF_254_ plates. Mass spectrometry analysis (LC-ESI-MS) was performed on a Bruker micro-TOF and with an Agilent 6430 T-Quadrupole spectrometer. High-resolution mass spectrometry (ESI-HRMS) was determined at the EPSRC National Mass Spectrometry facility at Swansea University (Swansea, UK).

### 3.2. General Radiochemistry: Source, Equipment and Analytical Methods

[^18^F]Fluoride was produced in an IBA Cyclon 18/9 cyclotron using the ^18^O(p,n)^18^F nuclear reaction. ^18^O-Enriched water (enrichment grade 98%, 2.2 mL, Nukem GmbH, Alzenau, Germany) was irradiated with 18 MeV protons. Radiofluorinations were performed on an Eckert & Ziegler module system. The drying procedures were performed with a vacuum pump N820 (Neuberger, Freiburg, Germany). Semi-prep HPLC (Phenomenex Synergi 4μ Hydro-RP 80, C-18, 10 × 250 mm) with a smartline pump 100–126 connected to the Eckert & Ziegler module system was used for the purification of the radiolabelled products. QMA and Al sep-pak (Waters corp., Milford, MA, USA) were used for purification of fluorine-18 intermediates. HPLC analytical evaluation was conducted on an Agilent Technology 1200 Series System with an analytical reversed phase column (Phenomenex Synergi 4μ Hydro-RP 80, C-18, 4.6 × 250 mm) coupled with a RAM/RAM Model 4 detector (Lablogic System, Ltd., Sheffield, UK) for radio-HPLC purposes.

### 3.3. Procedures and Spectroscopic Data for the Synthesis of the [^18^F]FLT-ProTide

Synthesis of *(2S)-ethyl-2-((chloro(phenyl)phosphoryl)amino)propanoate* (**10**). C_11_H_15_ClNO_4_P; MW: 291.6. Compound 10 was synthesized according to standard procedure [19]. Anhydrous triethylamine (2 eq; 0.662 mL; 0.480 g; 4.74 mmol) was added to the phenyldichlorophosphate **(9)** (1 eq; 0.354 mL; 0.500 g; 2.37 mmol) and l-alanine ethyl ester hydrochloride salt (1 eq; 0.364 g; 2.37 mmol) in anhydrous CH_2_Cl_2_ (5 mL) to obtain the final product 10 as a yellowish oil that was used without further purification. Yield: 92%. ^1^H NMR (500 MHz, CDCl_3_): δ 7.35–7.41 (m, 2H, Ar-H), 7.21–7.30 (m, 3H, Ar-H), 4.53 (m, 1H, NH), 4.21 (m, 1H, CH), 3.95 (m, 2H, CH_2_), 1.51 (m, 3H, CH_3_), 1.23 (m, 3H, CH_3_). ^31^P NMR (202 MHz, CDCl_3_): δ 7.71, 8.05. Spectroscopic data in agreement with literature [29,30,31].

Synthesis of *(2S)-ethyl2-(((((2R,3S,5R)-3-fluoro-5-(5-methyl-2,4-dioxo-3,4-dihydropyrimidin-1(2H)-yl)tetrahydrofuran-2-yl)methoxy)(phenoxy)phosphoryl)amino)propanoate* (**11**). C_21_H_27_FN_3_O_8_P, MW: 499.4. FLT (**8**) (1 eq; 0.100 g; 0.41 mmol) in anhydrous THF, was reacted with ^t^BuMgCl (1.5 eq; 0.08 mL) under nitrogen atmosphere. The reaction mixture was stirred at rt for 30 min. A solution of the phosphorochloridate **10** (2 eq; 0.239 g; 0.82 mmol) in anhydrous THF was then added dropwise and the reaction mixture was left to stir overnight. The solvent was then evaporated under reduced pressure and the residue was purified by silica gel column chromatography (CH_2_Cl_2/_CH_3_OH from 100% CH_2_Cl_2_ to 95% CH_2_Cl_2_) to give the FLT ProTide **(11)** as a yellowish oil. Yield: 23.5%. Rf: 0.44 in 90% CH_2_Cl_2_/10% CH_3_OH TLC system. ^1^H NMR (500 MHz, CDCl_3_): δ 8.60 (br s, 1H, NH), 7.55 (s, 1H, H-6), 7.33–7.41 (m, 2H, Ar-H), 7.20–7.29 (m, 3H, Ar-H), 6.23–6.30 (m, 1H, H-1′), 5.33 (m, 1H, H-3′), 4.43–4.53 (m, 2H, NH, H-4′), 4.21 (m, 1H, CH), 3.95 (m, 2H, CH_2_), 3.35-3.51 (m, 2H, H-5′, H-5”), 2.48–2.51 (m, 1H, H-2”), 2.55–2.33 (m, 1H, H-2′), 1.93–1.74 (m, 3H, CH_3_, thy), 1.30–1.18 (d, J = 6.9, CH_3_-ala). ^19^F NMR (479 MHz, CDCl_3_): δ −173.70, −175.20. ^31^P NMR (202 MHz, CDCl_3_): δ 4.34, 4.12. MS (ESI^+^): 498.1 [M − H^+^]. HPLC: Rt: 10.8 min; Purity > 96%; [Gradient: (0′) 95%H_2_O/5% CH_3_CN − (5′) 50% H_2_O/50% CH_3_CN − (15′) 50% H_2_O/50% CH_3_CN − (20′) 95% H_2_O/5% CH_3_CN]. Spectroscopic data in agreement with literature [17].

Synthesis of *(3R,5R)-3-(hydroxymethyl)-8-methyl-2,3-dihydro-5H,9H-2,5-methanopyrimido[2,1-b*][1,5,3]*dioxazepin-9-one* (**12**). MF: C_10_H_12_N_2_O_4_; MW: 224.22. Thymidine (**3**) (1 eq, 0.250g, 1.32mmol) and triphenylphosphine (Ph_3_P) (2 eq, 0.541 g, 2.64 mmol) were suspended in anhydrous acetonitrile (20 mL) and cooled down to −15 °C. Diisopropylazadicarboxylate (DIAD) (2 eq, 0.406 mL, 0.417 g, 2.64 mmol) was then added dropwise maintaining the temperature below −5 °C with vigorous stirring. The reaction was allowed to stir for 5h at 0 °C and then again cooled down to −20 °C. Cold ethyl acetate (20 mL) was added and the reaction was stirred for a further 15 min. A white precipitate was formed and was collected by Buchner filtration. The filtrate was washed with cold ethyl acetate and evaporated to dryness. The resulting crude compound was purified by silica gel column chromatography using 90% CH_2_Cl_2/_10% CH_3_OH as eluent to obtain the product **12** as a white solid. Yield: 52%. Rf: 0.5. ^1^H NMR (500 MHz, DMSO-*d_6_*): δ 7.55 (d, *J* = 1.2, 1H, ArH), 5.80 (d, *J* = 3.9, 1H, H-1′), 5.23 (brs, 1H, H-3′), 5.01 (t, 1H, 5′-OH), 4.20 (m, 1H, H-4′), 3.51 (m, 2H, H-5′, H-5”), 2.55 (d, *J* = 1.2, H-2′,1H), 2.47 (ddd, *J*^1,8^ = 19.0, *J*^1,4^ = 6.7, *J*^1,2^ = 3.0, 1H, H-2”), 1.76 (d, *J* = 1.1, 3H, CH_3_). Spectroscopic data in agreement with literature [32].

Synthesis of *1-((2R,4R,5R)-4-hydroxy-5-(hydroxymethyl)tetrahydrofuran-2-yl)-5-methylpyrimidine-2,4(1H,3H)-dione* (**13**). MF: C_10_H_14_N_2_O_5_; MW: 242. 3′-anhydrothymidine (**12**) (0.200 g; 0.892 mmol) in aq. 1.5 M NaOH (3.33 mL) was stirred in methanol (30 mL) under reflux for 3 h. Upon heating, the solution changed colour from clear to golden-brown. The reaction was monitored by TLC chromatography. When the conversion into the final product was confirmed, the solvent was evaporated under reduced pressure. The resulting crude compound was purified by silica gel column chromatography (CH_2_Cl_2/_CH_3_OH gradient from 100% to 90% of CH_2_Cl_2_) to obtain the final product (13) as a white powder. Yield: 64%. Rf: 0.4 in 90% CH_2_Cl_2_/10% CH_3_OH TLC system. ^1^H NMR (500 MHz, DMSO-*d_6_*): δ 11.24 (s, 1H, NH), 7.78 (s, 1H, H-6), 6.07 (dd, *J* = 8.5, 2.44, 1H, H-1′), 5.25 (d, *J* = 3.35, 1H, 3′-OH), 4.67 (t, *J* = 5.49, 1H, 5′-OH), 4.23 (m, 1H, H-3′), 3.60–3.84 (m, 3H, H-4′, H-5′ and H-5”), 2.55–2.59 (m, 1H, H-2”), 1.84 (dd, *J* = 14.95, *J* = 2.14, 1H, H-2′), 1.76 (s, 3H, CH_3_). Spectroscopic data in agreement with literature [32].

Synthesis of *((2S)-ethyl-2-(((((2R,3R,5R)-3-hydroxy-5-(5-methyl-2,4-dioxo-3,4-dihydropyrimidin-1(2H)-yl)tetrahydrofuran-2-yl)methoxy)(phenoxy)phosphoryl)amino)propanoate* (**14**). MF: C_21_H_28_N_3_O_9_P; MW: 497.4. Compound **14** was synthesised according to standard procedure [16]. 1-(2-deoxy-β-lyxofuranoxyl thymidine) (**13**) (1 eq; 0.175 g; 0.721 mmol) was reacted with NMI (5 eq; 0.287 mL; 0.297 g, 3.62 mmol) and ethyl-(2-chloro(phenyl)phosphorylamino)propanoate (**10**) (3 eq; 0.633 g; 2.17 mmol) to obtain a crude residue that was purified by silica gel column chromatography (97% CH_2_Cl_2/_3% CH_3_OH) to afford the final compound **14** as a white solid. Yield: 18.4%. Rf: 0.4. ^1^H NMR (500 MHz, CDCl_3_): δ 7.58 (d, J = 7.0, 1H, H-6), 7.35–7.43 (m, 2H, Ar-H), 7.20–7.29 (m, 3H, Ar-H), 6.25–6.32 (m, 1H, H-1′), 5.22 (m, 1H, 3′-OH), 4.94 (m, 1H, H-3′), 4.31–4.52 (m, 2H, NH, H-4′), 3.95–4.03 (m, 1H, CH), 3.68 (d, J = 7.0, 2H, CH_2_-ester), 3.35 (d, J = 16.0, 2H, H-5′, H-5”), 2.48–2.51 (m, 1H, H-2”), 2.09–2.25 (m, 1H, H-2′), 1.85 (d, J = 10.1, 3H, CH_3_-thy), 1.23 (m, 3H, CH_3_-ala), 1.16 (d, J = 7.2, 3H, CH_3_-ester). ^31^P NMR (200 MHz, CDCl_3_): δ 5.34, 4.98.

Synthesis of *(2S)-ethyl-2-(((((2R,3R,5R)-5-(5-methyl-2,4-dioxo-3,4-dihydropyrimidin-1(2H)-yl)-3-((methylsulfonyl)oxy)tetrahydrofuran-2-yl)methoxy)(phenoxy)phosphoryl)amino)propanoate* (**4**). MF: C_22_H_30_N_3_O_11_PS; MW: 575.5. Triethylamine (10 eq; 1.06 mL; 0.769 g; 7.6 mmol) and mesyl chloride (4 eq; 0.235 mL; 0.348 g; 3.04 mmol) were reacted with a solution of compound **14** (1 eq; 0.378 g; 0.76 mmol) in anhydrous CH_2_Cl_2_ (20 mL) at 0 °C. The reaction mixture was stirred at 0 °C for 10 min and then warmed to rt and stirred for 1.5 h. The crude mixture was diluted with sat. NaHCO_3_ solution and extracted with CH_2_Cl_2_. After drying over Na_2_SO_4_, the solution was reduced under reduced pressure and the resulting crude compound was purified by silica gel column chromatography (CH_2_Cl_2/_CH_3_OH gradient from 100% CH_2_Cl_2_ to 95% CH_2_Cl_2_) to give the product **4** as a white solid. Yield: 29.5%. Rf: 0.45 in 90% CH_2_Cl_2/_10% CH_3_OH TLC system. ^1^H NMR (500 MHz, CDCl_3_): δ 8.93–8.96 (s, 1H, NH, thy), 7.33–7.32 (d, J = 7.0, 1H, H-6), 7.24–7.28 (m, 2H, Ar-H), 7.10–7.15 (m, 3H, Ar-H), 6.23–6.21 (m, 2H, H-1′), 5.19–5.15 (s, 1H, NH-ala), 3.84–4.37 (m, 7H, H-3′, H-4′, CH, H-5′, H-5”, CH_2_-ethyl), 2.96–3.01 (s, 3H, SO_2_CH_3_), 2.72–2.75 (m, 1H, H-2”), 2.38–2.42 (m, 1H, H-2′), 1.87 (d, J = 1.2, 3H, CH_3_-thy), 1.28–1.33 (m, 3H, CH_3_-ala), 1.19–1.16 (m, 3H, CH_3_-ethyl). ^13^C NMR (125 MHz, CDCl_3_) δ 173.7–173.4 (C=O, acetyl), 163.6 (C-2), 150.42 (C-1), 135.1 (C-4), 129.8 (C-2; C-6Ar), 125.2 (C-4Ar), 120.2 (C-3; C-5Ar), 111.6 (C-3), 83.5 (C-1′), 79.9 (C-3′), 77.3 (C-4′), 63.7 (C-5′), 61.7 (CH_2_-ethyl), 50.8 (CH-ala), 39.2 (C-2′), 38.8 (CH_3_-mesyl), 21.34 (CH_3_-ethyl), 14.04 (CH_3_-ala), 12.76 (CH_3_-thy). ^31^P NMR (202 MHz, CDCl_3_): δ 2.92, 2.63. MS(ESI^+^): 576.2 [M + H^+^]. HPLC: Rt: 13.2 min; Purity > 98%; [Gradient: (0′) 95% H_2_O/5% CH_3_CN − (5′) 50% H_2_O/50% CH_3_CN − (15′) 50% H_2_O/50% CH_3_CN − (20′) 95% H_2_O/5% CH_3_CN].

Synthesis of *(2S)-ethyl-2-(((((2R,3R,5R)-5-(5-methyl-2,4-dioxo-3,4-dihydropyrimidin-1(2H)-yl)-3-(tosyloxy)tetrahydrofuran-2-yl)methoxy)(phenoxy)phosphoryl)amino)propanoate* (**5**). MF: C_28_H_34_N_3_O_11_PS; MW: 651.6. To a solution of compound **14** (1 eq; 0.181 g; 0.364 mmol) in pyridine (5 mL), tosyl chloride (2 eq; 0.138 g; 0.727 mmol) and silver trifluoromethanesulfonate (AgOTf) (2 eq; 0.186 g; 0.727 mmol) were added at 0 °C. The reaction was stirred for 1 h and then slowly allowed to warm to rt and stirred for another 2 h. The reaction mixture was then diluted with EtOAc, filtered, and the filtrate was washed with H_2_O and brine. The organic layer was dried over anhydrous Na_2_SO_4_ and the solvent was evaporated under reduced pressure. The crude residue was purified by silica gel column chromatography (95% CH_2_Cl_2/_5% CH_3_OH) to furnish the tosylated compound 5 as a yellowish solid. Yield: 35%. Rf: 0.55. ^1^H NMR (500 MHz, CDCl_3_) δ 7.76–7.75 (d, J = 1.9, 1H, H6 Ar), 7.34 (dd, J = 15.7, 8.6, 4H, Ar-H tosyl), 7.27–7.17 (m, 5H, Ar), 6.19–5.20 (td, J = 7.8, 2.9, 1H, H-1′), 4.45–3.74 (m, 10H), 2.72–2.61 (m, 1H, CH-ala), 2.46 (s, 3H, CH_3_, tosyl), 1.85 (s, 3H, CH_3_, thy), 1.39 (t, J = 7.2 Hz, 3H, CH_3_, ethyl), 1.31 (m, 3H, CH_3_-ala). ^13^C NMR (126 MHz, CDCl_3_) δ 173.65, 173.59 (C-ala), 163.52 (C1-thy), 150.58, 150.53 (C3-thy), 150.25, 150.19 (C1-tosyl), 145.99, 145.98 (C1-phenyl), 135.04, 134.95 (CH-thy), 133.05, 132.90 (C4-tosyl), 130.27, 130.21 (CH, C2, C6-tosyl), 129.75, 129.70 (CH, C2, C6-phenyl), 127.64, 127.58 (CH, C4-phenyl), 125.10, 120.35 (CH, C3, C5-phenyl), 120.31, 120.21 (CH, C3, C5-tosyl), 111.15, 110.98 (C3-thy), 84.22, 83.99 (CH, C1′), 80.96, 80.90 (CH, C3′), 80.72, 80.66 (CH, C4′), 63.89, 63.85 (CH_2_, C5′), 63.33, 63.29 (CH_2_, ethyl), 50.39, 50.38 (CH, ala), 39.03 (CH_2_, C3′), 21.69, 21.00 (CH_3_-ethyl), 20.96, 20.95 (CH_3_-tosyl), 14.12 (CH_3_, ala), 12.49, 12.44 (CH_3_-thy). ^31^P NMR (202 MHz, CDCl_3_) δ 2.78, 2.66. MS (ESI)^+^: 652.2 [M + H^+^]; 674.1 [M + Na^+^]. HPLC: Rt: 16.03 min; Purity > 98%; [Gradient: (0′) 95% H_2_O/5% CH_3_CN − (5′) 50% H_2_O/50% CH_3_CN − (15′) 50% H_2_O/50% CH_3_CN − (20′) 95% H_2_O/5% CH_3_CN].

Synthesis of *(S)-ethyl-2-(((((2R,3R,5R)-5-(5-methyl-2,4-dioxo-3,4-dihydropyrimidin-1(2H)-yl)-3-(((4-nitrophenyl)sulfonyl)oxy)tetrahydrofuran-2yl)methoxy)(phenoxy)phosphoryl)amino)propanoate* (**6**). MF: C_27_H_31_N_4_O_13_PS; MW: 682.5. The ProTide **14** (1 eq; 1.16 g; 2.34 mmol) was dissolved in pyridine (20 mL) at 0 °C. 4-nitrobenzenesulfonylchloride (nosyl chloride) (2 eq; 1.06 g; 4.79 mmol) and silver trifluoromethanesulfonate (AgOTf) (2 eq; 1.23 g; 4.79 mmol) were added and the reaction mixture was stirred at 0 °C. After 1h the reaction mixture was allowed to slowly warm to rt and stirred for another 2 h. The reaction mixture was then diluted with EtOAc, filtered, and the filtrate was washed with H_2_O and brine. The organic layer was dried over anhydrous Na_2_SO_4_ and the solvent was evaporated under reduced pressure. Purification of the crude residue was accomplished by silica gel column chromatography (95% CH_2_Cl_2_/5% CH_3_OH) to give the desired compound **6** as a yellowish solid. Yield: 60%. Rf: 0.6. ^1^H NMR (500 MHz, CDCl_3_) δ 8.77–8.67 (s, 1H, NH, thy), 8.41–8.39 (d, J = 2.2, 2H-Ar, nosyl), 8.14–8.08 (m, 2H-Ar, nosyl), 7.71 (ddd, J = 7.6, 4.7, 1.7, 1H, H6), 7.42–7.30 (m, 5H-Ar), 6.28–5.28 (m, 1H-H-1′), 4.51–3.75 (m, 7H), 2.79–2.46 (m, 1H, CH-ala), 1.96–1.86 (m, 2H, H-2′, H-2′’), 1.37 (t, J = 7.6, 3H, CH_3_ ester), 1.31–1.25 (m, 6H, CH_3_-thy, CH_3_-ala). ^13^C NMR (126 MHz, CDCl_3_) δ 173.66, 173.36 (C-ala), 163.48 (C2, thy), 151.13, 151.10 (C1, thy), 150.29, 150.25 (C1, nosyl), 141.45, 141.35 (C1, phenyl), 134.73, 134.67 (CH, thy), 129.86, 129.81 (CH, C2-6, nosyl), 129.15, 129.13 (CH, C2, C6, phenyl), 125.28 (CH, C4, phenyl), 124.84, 124.77 (CH, C3, C5, phenyl), 120.15, 120.11 (CH, C3, C5, nosyl), 120.06, 120.02, 111.36, 111.23 (C, C3, thy), 84.14, 84.00 (CH, C1′), 80.58, 80.52 (CH, C3′), 80.25, 80.18 (CH, C4′), 63.17, 63.14 (CH_2_, C5′), 62.82, 62.79 (CH_2_, ethyl), 50.41, 50.20 (CH, ala), 39.18, 39.16 (CH_2_, C2′), 20.94, 20.90 (CH_3_, ethyl), 14.12, 14.11 (CH_3_, ala), 12.58, 12.56 (CH_3_, thy). ^31^P NMR (202 MHz, CDCl_3_) δ 2.75, 2.48. MS (ESI)^+^: 705.1 [M + Na^+^]. HPLC: Rt: 15.88 min; Purity > 99%; [Gradient: (0′) 95% H_2_O/5% CH_3_CN − (5′) 50% H_2_O/50% CH_3_CN − (15′) 50% H_2_O/50% CH_3_CN − (20′) 95% H_2_O/5% CH_3_CN].

Synthesis of *tert-butyl-3-((2R,4R,5R)-5-(((((tert-butoxycarbonyl)((S)-1-ethoxy-1-oxopropan-2-yl)amino)(phenoxy)phosphoryl)oxy)methyl)-4-(((4-nitrophenyl)sulfonyl)oxy)tetrahydrofuran-2-yl)-5-methyl-2,6-dioxo-3,6-dihydropyrimidine-1(2H)-carboxylate* (**7**). MF: C_37_H_47_N_4_O_17_PS. MW: 882.83. The nosylated ProTide (**6**) (1 eq, 0.050 g, 0.073 mmol) was dissolved in pyridine (6 mL) at rt under nitrogen atmosphere. To the stirring solution, di(tert-butyl)dicarbonate (Boc_2_O) (1.3 eq, 0.021 mL, 0.020 g, 0.095 mmol) was added dropwise and the reaction was stirred for 16h. The crude mixture was evaporated under reduced pressure and was purified by silica gel column chromatography (CH_2_Cl_2/_CH_3_OH gradient from 100% CH_2_Cl_2_ to 95% CH_2_Cl_2_) to give the final di-protected nosylated derivate **7** as a yellowish oil. Yield: 56%. Rf: 0.67 in 90% CH_2_Cl_2/_10% CH_3_OH as TLC system. ^1^H NMR (500 MHz, CDCl_3_) δ 8.47–8.36 (d, 2H, J = 2.2, Ar, nosyl), 8.17–8.08 (m, 2H, Ar, nosyl), 7.38 (m, 1H, Ar), 7.32–7.24 (m, 4H), 7.17 (s, 1H, thy), 6.31–6.24 (m, 1H-H-1′), 4.51–3.75 (m, 7H), 2.78–2.43 (m, 1H, CH-Ala), 2.31–2.23 (m, 1H-H-2′), 2.01 (m, 3H, CH_3_, thy), 1.53–1.49 (m, 9H, CH_3_, tert-butyl), 1.44 (m, 9H, CH_3_, tert-butyl), 1.38 (t, J = 7.6, 3H, CH_3_, ester), 1.31 (m, 3H, CH_3_, ala). ^13^C NMR (126 MHz, CDCl_3_) δ 175.50, 174.29 (C, ala), 161.32 (C2, thy), 150.13, 150.08 (C1, thy), 150.02, 150.00 (C1, nosyl) 143.51, 142.21 (C1, phenyl), 132.71, 132.23 (CH, thy), 130.68, 129.99 (CH, C2–C6, nosyl), 129.34, 129.5 (CH, C2, C6, phenyl), 126.28 (CH, C4, phenyl), 125.79, 124.85 (CH, C3, C5, phenyl), 120.15, 120.13 (CH, C3,C5, nosyl), 120.06, 120.02, 111.36, 111.23 (C, C3, thy), 84.13, 84.10 (CH, C1′), 80.78–80.77 (C-tert-butyl), 80.58, 80.52 (CH, C3′), 80.25, 80.18 (CH, C4′), 63.21, 63.20 (CH_2_, C5′), 62.79, 62.77 (CH_2_, ethyl), 50.39, 50.30 (CH, ala), 39.18, 39.15 (CH_2_, C2′), 28.41–28.23 (CH_3_, tert-butyl), 20.94, 20.90 (CH_3_, ethyl), 14.12, 14.11 (CH_3_, ala), 12.58, 12.56 (CH_3_, thy). ^31^P NMR: (202 MHz, CDCl_3_): δ 2.61, 2.53. MS (ESI^+^): 905.83 [M + Na^+^]. HPLC: Rt: 17.1 min; Purity > 99%; [Gradient: (0′) 95% H_2_O/5% CH_3_CN − (5′) 50% H_2_O/50% CH_3_CN − (15′) 50% H_2_O/50% CH_3_CN − (20′) 95% H_2_O/5% CH_3_CN].

Synthesis of *tert-butyl-3-((2R,4S,5R)-5-((((N-((S)-1-ethoxy-1-oxopropan-2-yl)-3,3-dimethylbutanamido)(phenoxy)phosphoryl)oxy)methyl)-4-[^18^F]fluoro-tetrahydrofuran-2-yl)-5-methyl-2,6-dioxo-3,6-dihydropyrimidine-1(2H)-carboxylate* (**15**). MF: C_32_H_45_^18^FN_3_O_11_P; MW: 696.70. Aqueous [^18^F]fluoride (2–8 GBq) produced by the cyclotron was trapped in a QMA cartridge and was then eluted through the cartridge by an aqueous solution of KHCO_3_ and Kryptofix in CH_3_CN. The resulting [^18^F]F^-^/KHCO_3_/Kryptofix complex was dried by an azeotropic distillation with anhydrous CH_3_CN (2 × 1 mL) under reduced pressure and a stream of nitrogen. A solution of the precursor **7** (10 mg) in anhydrous CH_3_CN (1 mL) was added and the reaction was stirred for 30 min at 95 °C. The resulting reaction mixture was passed through an alumina cartridge to obtain the radiolabelled product **15**. The reaction mixture was analysed by analytical radio HPLC: Rt: 15 min (analytical HPLC: (0′) 95% H_2_O/5% CH_3_CN − (5′) 50% H_2_O/50% CH_3_CN − (15′) 50% H_2_O/50% CH_3_CN − (20′) 95% H_2_O/5% CH_3_CN).

Synthesis of *ethyl ((((2R,3S,5R)-3-[^18^F]fluoro-5-(5-methyl-2,4-dioxo-3,4-dihydropyrimidin-1(2H)-yl)tetrahydrofuran-2-yl)methoxy)(phenoxy)phosphoryl)-l-alaninate* (**1**). MF: C_21_H_27_^18^FN_3_O_8_P; MW: 498.43. To the Boc protected [^18^F]-radiolabelled ProTide (**15**), a solution of 2 N HCl (1 mL) was added and the reaction was stirred for 10 min. The solution was then neutralised with 2 N NaOH (1 mL). The crude mixture was then purified by semi-preparative HPLC and the desired compound was eluted after 35 min at a flow rate of 3.5 mL/min using 70% H_2_O/30% CH_3_CN as mobile phase. The compound was then dried under a stream of nitrogen, taken up in saline and flushed through a sterility filter to obtain the aqueous solution of [^18^F]FLT ProTide (**1**). RCY of 15–30% (n = 5, decay-corrected from end of bombardment (EoB)), with high radiochemical purities (97%) and molar activities of 56 GBq/μmol. The total synthesis time was 130 min after the end of bombardment (EoB). The reaction mixture was analyzed by analytical radio-HPLC. Analytical HPLC: (0′) 95% H_2_O/5% CH_3_CN − (5′) 50% H_2_O/50% CH_3_CN − (15′) 50% H_2_O/50% CH_3_CN − (20′) 95% H_2_O/5% CH_3_CN); Rt: 9.5 min.

### 3.4. Procedures and Analytical Data for the Synthesis of the [^18^F]FIAU ProTide

Synthesis of *2′-deoxy-2′-α-fluoro-5-iodouridine* (**19**). MW: 372.1; MF: C_9_H_10_FIN_2_O_4_. Iodine (1.2 eq; 1.24 g; 4.87 mmol) and ceric ammonium nitrate (CAN) (1 eq; 2.23 g; 4.062 mmol) were added to a stirring solution of 2′-β-fluoro-2′-deoxyuridine (**18**) (2 eq; 2.0 g; 8.12 mmol) in anhydrous acetonitrile (50 mL). The mixture was stirred at 75 °C for 1 h and was then quenched with a saturated solution of Na_2_S_2_O_3_ and concentrated under reduced pressure. The residue was then re-dissolved in ethyl acetate and washed twice with saturated NaCl. The organic layer was dried over MgSO_4_, filtered and concentrated to give compound **19** as a pale yellow solid. Yield: 60%. HPLC: Rt: 2.3 min; Purity > 95% (98% H_2_O/2% CH_3_CN). ^1^H NMR (500 MHz, DMSO-*d_6_*): δ 11.69 (s, 1H, NH), 8.53 (s, 1H, 6-CH), 5.86 (d, *J* = 15.8, 1H, 1’-CH), 5.60 (d, *J* = 6.4, 1H, 3’-OH), 5.39 (t, *J* = 4.5, 1H, 3’-CH), 5.04 (dd, *J* = 5.2, 4.1, 1H, 2’-CH), 4.18 (ddd, *J* = 23.4, 11.4, 7.2, 1H, 4’-CH), 3.90 (d, *J* = 8.2, 1H, 5’-OH), 3.85–3.79 (m, 1H, 5’-CH), 3.63–3.58 (m, 1H, 5’-CH). ^13^C NMR (125 MHz, DMSO-*d_6_*): δ 167.88 (C=O), 165.01 (C=O), 145.02 (C-6), 125.19 (CH, C-2’), 121.26 (CH, C-1’), 115.81 (CH, C-4’), 61.11 (CH, C-5), 57.30 (CH, C-3’), 45.87 (CH, C-5’). ^19^F NMR (470 MHz, DMSO-*d_6_*): δ −202.09. Spectroscopic data in agreement with literature [10].

Synthesis of *benzyl(chloro(phenoxy)phosphoryl)-L-alaninate* (**20**). MW: 353.73; MF: C_16_H_17_ClNO_4_P. Compound **20** was synthesised according to the standard procedure [16]. Anhydrous triethylamine (2 eq; 1.26 mL; 0.918 g; 9.08 mmol), phenyl dichlorophosphate (1 eq; 0.678 mL; 0.958 g; 4.54 mmol) and L-alanine benzyl ester hydrochloride salt (1 eq; 1.50 g; 4.54 mmol) were reacted to give compound **20** as a yellowish oil. Yield: 88%. ^1^H NMR (500 MHz, CDCl_3_): δ 7.54–7.47 (m, 7H, Ar-H), 7.46–7.40 (m, 3H, Ar-H), 5.27 (d, *J* = 8.4, 2H, CH_2_-ester), 4.69 (d, *J* = 9.9, 1H, NH), 4.13 (dd, *J* = 34.4, 29.8, 1H, CH-ala), 1.52 (m, 3H, CH_3_-ala). ^31^P NMR (202 MHz, CDCl_3_): δ 8.03, 7.75. Spectroscopic data in agreement with literature [29,30,31].

Synthesis of *benzyl((((2R,3R,4S,5R)-4-fluoro-3-hydroxy-5-(5-iodo-2,4-dioxo-3,4-dihydropyrimidin-1(2H)-yl)tetrahydrofuran-2-yl)methoxy)(phenoxy)phosphoryl)-l-alaninate* (**21**). MF: C_25_H_26_FIN_3_O_9_P; MW: 689.3. Compound **21** was prepared according to the standard procedure [16]. 1-(3-Fluoro-4-hydroxy-5-(hydromethyl)tetrahydrofuran-2-yl)-5-iodopyrimidine-2,4(1H,3H)-dione (**19**) (1 eq; 0.400 g; 1.07 mmol) and NMI (5 eq; 0.424 mL; 0.439 g; 5.35 mmol) were reacted with benzyl 2-(chloro(phenoxy)phosphorylamino)propanoate (3 eq; 1.05 g; 3.22 mmol) (**20**). The crude mixture was purified by silica gel column chromatography (CH_2_Cl_2/_CH_3_OH from 100% CH_2_Cl_2_ to 95% CH_2_Cl_2_) to obtain the product **21** as a yellowish oil. Yield: 10%. ^1^H NMR (500 MHz, CDCl_3_): δ 10.59 (s, 1H, 3-NH), 7.89 (s, 1H, 6-CH), 7.53–7.48 (m, 2H, CH-phenyl), 7.45 (t, J = 8.0, 2H, CH-phenyl), 7.41–7.38 (m, 2H, CH-benz), 7.17–7.15 (m, 2H, CH-benz), 7.13 (t, J = 8.0, 1H, CH-benzyl), 7.12 (t, J = 7.4, 1H, CH-phenyl), 5.99 (m, 1H, 1’-CH), 5.79 (dd, J = 47.6, 4.6, 1H, 2’-CH), 5.14 (m, 2H, CH_2_-benz), 4.90 (m, 2H, 5’-CH_2_), 4.39 (m, 1H, 4’-CH), 4.27 (m, 1H, 3’-CH), 4.19 (m, 1H, 3’-OH), 4.02 (m, 1H, NH-ala), 3.99 (m, 1H, CH-ala), 1.29 (d, J = 7.0, 3H, CH_3_-ala). ^13^C NMR (125 MHz, CDCl_3_): δ 171.9 (C=O, ala), 168.12 (C=O, C-4), 144.18 (C=O, C-2), 149.0 (C-phenyl), 147.91 (C-benzyl), 145.5, 145.1 (CH, C-6), 128.31–121.48 (CH, Ar-C), 94.3 (C-5), 92.10 (CH, C-4’), 89.13 (CH, C-2’), 83.6, 82.97 (CH, C-1’), 81.08, 80.97 (CH_2,_ C-ester), 69.61, 68.14 (CH, C-3’), 67.23 (CH, C-5’), 52.8 (CH-ala), 21.98 (CH_3_-ala). ^19^F NMR (470 MHz, CDCl_3_): δ -200.91, -201.15. ^31^P NMR (202 MHz, CDCl_3_): δ 3.90, 3.86. MS (ESI)^+^: 690.3 [M + H^+^]. HPLC: Rt: 13.4 min; purity > 97%; [Gradient: (0′) 95% H_2_O/5% CH_3_CN − (5′) 50% H_2_O/50% CH_3_CN − (15′) 50% H_2_O/50% CH_3_CN − (20′) 95% H_2_O/5% CH_3_CN].

Synthesis of *(2R,3S,4R,5R)-5-((benzoyloxy)methyl)-3-[^18^F]fluoro-tetrahydrofuran-2,4-diyl-dibenzoate* (**17**). MF: C_26_H_21_^18^FO_7_ MW: 463.4 Aqueous [^18^F]fluoride (4.11 GBq), produced by the cyclotron was trapped in a QMA cartridge before it was eluted with an aqueous solution of KHCO_3_ and Kryptofix in anhydrous CH_3_CN. The [^18^F]F^-^/KHCO_3_/Kryptofix complex was dried by co-evaporation with anhydrous CH_3_CN (2 × 1 mL) under reduced pressure and a stream of nitrogen. A solution of the triflate precursor (**16**) (10 mg) in anhydrous CH_3_CN (1 mL) was added to the reaction vial and the reaction was stirred for 30 min at 95 °C. The mixture was passed through an alumina cartridge to obtain the radiolabelled product **17** that was used for next step without further purification. The reaction mixture was analysed by analytical HPLC. Rt: 8.3 min (100% H_2_O).

Synthesis of *5-iodo-2,4-bis((trimethylsilyl)oxy)pyrimidine* (**23**). MF: C_10_H_19_IN_2_O_2_Si_2_; MW: 382.3. To a solution of 5-iodouracil (**22**) (1 eq; 10 mg; 0.042 mmol) in dichloroethane (500 μL), hexamethyldisilazane (11.4 eq; 100 μL; 0.0774 mg; 0.479 mmol) and TMSOTf (13.1 eq; 100 μL, 0.123 mg; 0.549 mmol) were added. The mixture was stirred for 2 h at 85 °C and was used for next step without further purification. The purity of the compound was assessed by analytical HPLC (Rt = 6.9 min; 88% H_2_O/12% CH_3_CN) and LC-MS ([M + H^+^]: 383.2). Spectroscopic data was in agreement with literature [10].

Synthesis of *1-((2R,3S,4R,5R)-3-[^18^F]fluoro-4-hydroxy-5-(hydroxymethyl)tetrahydrofuran-2-yl)-5-iodopyrimidine-2,4(1H,3H)-dione ([^18^F]FIAU)* (**24**). MF: C_9_H_10_^18^FN_2_O_5_ MW: 371.0. Compound **17** was delivered to a vial containing 2-4-bis(trimethylsilyl)-5-iodouracil (**23**). The mixture was then heated at 85 °C for 60 min. To this mixture, 0.5 M of NaOCH_3_ in CH_3_OH (1 mL) was then added and the reaction was stirred at 85 °C for another 5 min. The precipitate was then reconstituted in water (1 mL) and neutralised with 6 N HCl. The reaction mixture was analyzed by analytical HPLC showing the formation of the 2 anomers (α and β) of the 2′-deoxy-2′-fluoro-5-iodouridine. Analytical HPLC: 98% H_2_O/2% CH_3_CN; Rt: α anomer 2.1 min; β anomer 2.9 min). The anomeric mixture was then purified by semi-preparative HPLC and the target compound (**24**) was eluted after 7.3 min at a flow rate of 3.5 mL/min using 20% CH_3_CN/80% H_2_O as mobile phase to obtain the final compound. HPLC: Rt: 2.1 min; 98% H_2_O/2% CH_3_CN. Data in agreement with literature [10].

Synthesis of *[^18^F]FIAU ProTide* (**2**). MF: C_25_H_26_^18^FIN_3_O_9_P; MW: 688.3. To [^18^F]FIAU (**24**), NMI (0.1 mL) and a solution of benzyl-2-(chloro(benzyloxy)phosphorylamino)propanoate (**20**) (0.050 g) in anhydrous THF (0.5 mL) were added together under nitrogen atmosphere. The reaction mixture was stirred at 50 °C for 20 min and then dried under a flow of nitrogen, re-dissolved in CH_3_CN and purified via semi-preparative HPLC. The compound **2** was eluted after 23 min at a flow rate of 3.5 mL/min using 50% CH_3_CN/50% H_2_O as the mobile phase. The solvent was then removed from the mixture under a stream of nitrogen. The final product was then re-formulated in saline and flushed through a sterility filter to furnish a clean sterile aqueous solution of [^18^F]FIAU ProTide (**2**). Radiochemical reactions were carried out using starting activities between 4–15 GBq, leading to RCY’s of 1–5% (*n* = 7, decay-corrected from end of bombardment (EoB)), with high radiochemical purities (98%) and molar activities of 53 GBq/μmol. The total synthesis time was 240 min after the end of bombardment (EoB). Analytical HPLC: (0′) 95% H_2_O/5% CH_3_CN - (5′) 50% H_2_O/50% CH_3_CN- (15′) 50% H_2_O/50% CH_3_CN- (20′) 95% H_2_O/5% CH_3_CN); Rt: 12.3 min.

## 4. Conclusions

Phosphoramidate ProTide technology is a successful prodrug strategy to deliver nucleosides to their target sites, reducing toxicity issues and improving the potency of their parent nucleosides. Several fluorinated ProTides are currently being evaluated as anticancer and antiviral agents at different stages of clinical trials. PET imaging has the potential to provide the pharmacokinetic profile of certain drug candidates directly in vivo and therefore to predict the response to therapy. In this study we have developed the first radiochemical synthesis of the [^18^F]FLT ProTide (**1**) chosen as a model standard of the class of 3′-fluorinated ProTides. An automated late stage [^18^F]fluorination was tested on four different precursors with the best yields obtained when using a di-Boc protected nosyl derivative (**7**). The late stage fluorination and easy purification make this tracer a good candidate as a PET imaging probe with substantial potential for clinical application.

[^18^F]FIAU ProTide (**2**) was synthesised as a model of the class of 2-‘fluorinated ProTides. Despite the early stage introduction of the fluorine-18, we have optimized the following steps involving the formation of the phosphoramidate bond. This optimization reduced the overall reaction time whilst maintaining a reasonable yield and high purity of the final compound. 

To our knowledge, this is the first time that [^18^F] radiolabelled ProTides have been synthesised. These radiotracers have the potential in preclinical models to further elucidate the in vivo mechanism of biodistribution and metabolism, as well as to be clinically translated for diagnostic and therapeutic evaluation purposes.

## Figures and Tables

**Table 1:** Tentative optimization of the glycosylation reaction.

Solvent	Temperature	Time	Catalyst	Ratio β:α
CH_3_CN	85 °C	30 min	TMSOTf	Incomplete
CH_3_CN	85 °C	45 min	TMSOTf	Incomplete
CH_3_CN	85 °C	1 h	TMSOTf	2:1
CH_3_CN	95 °C	30 min	TMSOTf	Incomplete
CH_3_CN	85 °C	15 min	TMSOTf+SnCl_4_ [24]	1:1.3

## Supplementary Materials

The following are available online. Figure S1. Internalization and metabolism of ProTides, bypassing the first-rate limiting step of the nucleoside analogues phosphorylation cascade; Figure S2. ^31^P NMR stability study; Figure S3: E&Z modular lab sketch; Figure S4: Representative analytical HPLC chromatogram for the fluorination of the mesyl precursor **4**; Figure S5: Representative analytical HPLC chromatogram for the fluorination of the tosyl precursor **5**; Figure S6: Representative analytical HPLC chromatogram for the fluorination of the nosyl unprotected precursor **6**; Figure S7: Representative analytical HPLC chromatogram of the fluorination of the nosyl protected precursor **7**; Figure S8: Representative analytical HPLC chromatogram for the deprotection of the precursor **15** before purification; Figure S9: Representative analytical HPLC chromatogram for the fluorination of the sugar; Figure S10: Representative analytical HPLC chromatogram of the glycosylation reaction; Figure S11: Representative analytical HPLC chromatogram of the coupling reaction; Table S1: Radiolabelling attempts for the mesyl precursor (compound **4**); Table S2: Radiolabelling attempts for the tosyl precursor (compound **5**); Table S3: Radiolabelling attempts for the unprotected nosyl precursor (compound **6**).
