# Supplementary material for: Radiosynthesis of [18F]-Labelled Pro-Nucleotides (ProTides)
Source: Molecules. 2020 Feb 6;25(3):704. doi: 10.3390/molecules25030704 (PMC7037993; doi:10.3390/molecules25030704)
Supplement: Supplementary file 1 [file molecules-25-00704-s001.pdf]

Radiosynthesis of [^{18}F]-Labelled Pro-Nucleotides (ProTides)

Alessandra Cavaliere[1, 2], Katrin C. Probst[2], Stephen Paisey[2], Christopher Marshall[2], Abdul K. H. Dheere[3], Franklin Aigbirhio[3], Christopher McGuigan[1] and Andrew D. Westwell[1,*]

Supplementary Materials

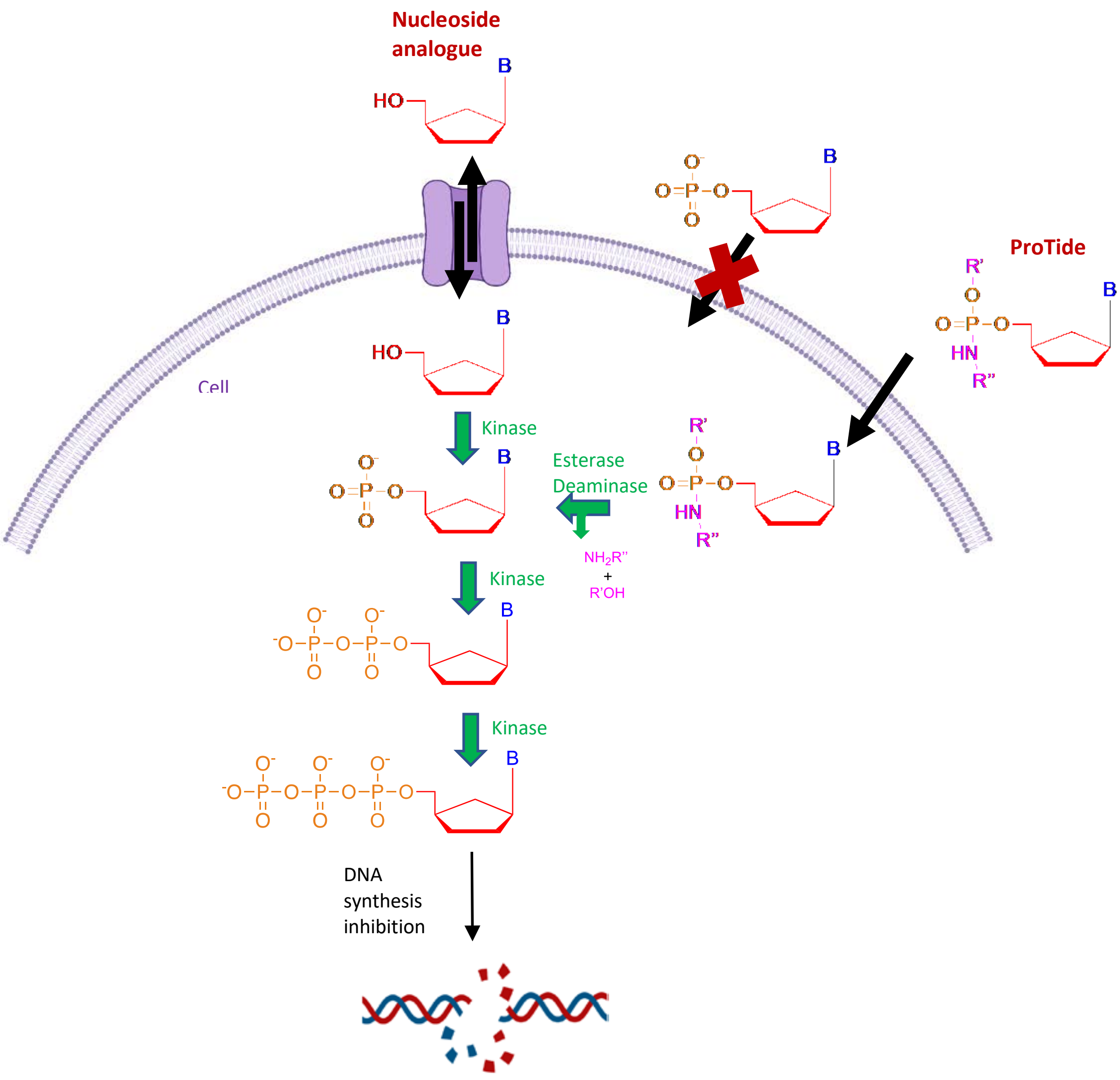

Figure **S1**: Internalization and metabolism of ProTides, bypassing the first-rate limiting step of the nucleoside analogues phosphorylation cascade.

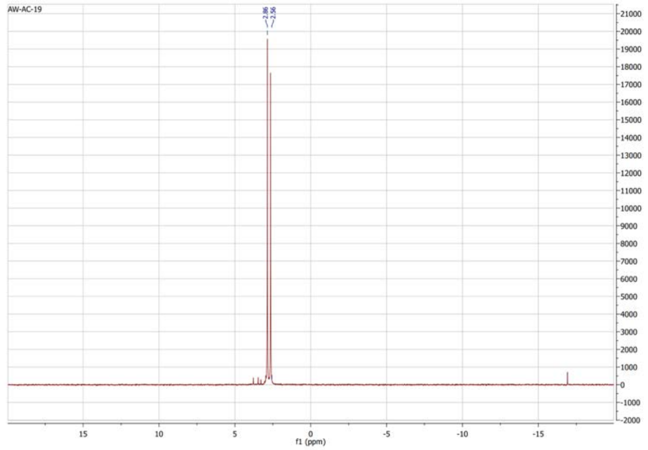

Figure S2. ^{31}P NMR stability study. Two characteristic peaks of the FLT ProTide diasteroisomeric mixture show the same chemical shift when compound **11** was heated at 120 °C over 1h, confirming the stability of the ProTide moiety.

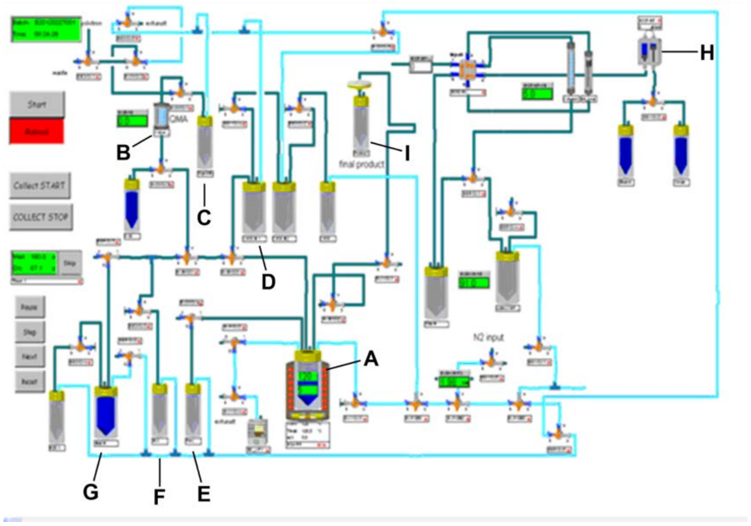

A. Reaction vial.
B. QMA cartridge preconditioned with 5 mL of an 8.4% aqueous solution of $NaHCO_3$ solution followed by 10 mL of water, to trap $^{18}F^-$ from the cyclotron.
C. Kryptofix [2.2.2] vial.
D. Anhydrous acetonitrile vial for the azeotropic evaporation.
E. Precursor vial filled with the precursor dissolved in the reaction solvent.
F. Acid vial filled with HCl for eventual deprotection.
G. Base vial filled with NaOH for eventual neutralization.
H. Vacuum pump for solvent removal.
I. Final product vessel for product isolation.

Figure S3: E&Z modular lab sketch.

Precursor	Solvent	mg	T(°C)	Time	($^{18}F^{-}$)	^{18}F-FLTProtide	^{18}F-by-products
**4**	DMF	10mg	120°C	15min	810 MBq	No	No
**4**	DMF	10mg	120°C	20min	2.35 GBq	No	No
**4**	DMF	10mg	120°C	30min	910 MBq	No	No
**4**	DMF	20 mg	120°C	15min	580 MBq	No	No
**4**	DMF	20 mg	120°C	20min	970 MBq	No	No

Table S1: Radiolabeling attempts for the mesyl precursor (compound **4**).

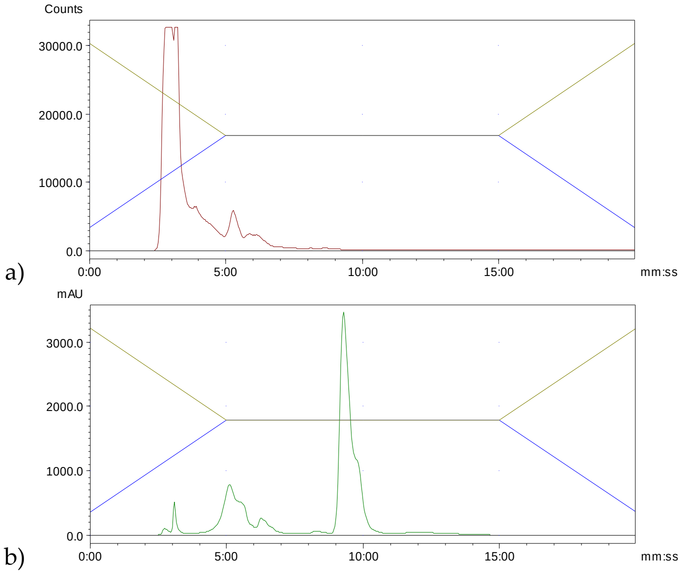

Figure S4: Representative analytical HPLC chromatogram for the attempted fluorination of the mesyl precursor **4.** a) Radiochromatogram showing mostly unreacted [^{18}F]fluoride; b) UV chromatogram showing mostly unreacted mesyl precursor.

Precursor	Solvent	Mg	T(°C)	Time	($^{18}F^{-}$)	^{18}F-FLTProtide	^{18}F-by-products
5	CH_3CN	10 mg	90°C	15min	2.0 GBq	No	Yes
5	CH_3CN	10 mg	90°C	20min	1.2 GBq	No	Yes
5	CH_3CN	10 mg	90°C	30min	2.5 GBq	No	Yes
5	DMF	10 mg	120°C	15min	2.3 GBq	No	No

Table S2: Radiolabeling attempts for the tosyl precursor (compound **5**).

a)

b)

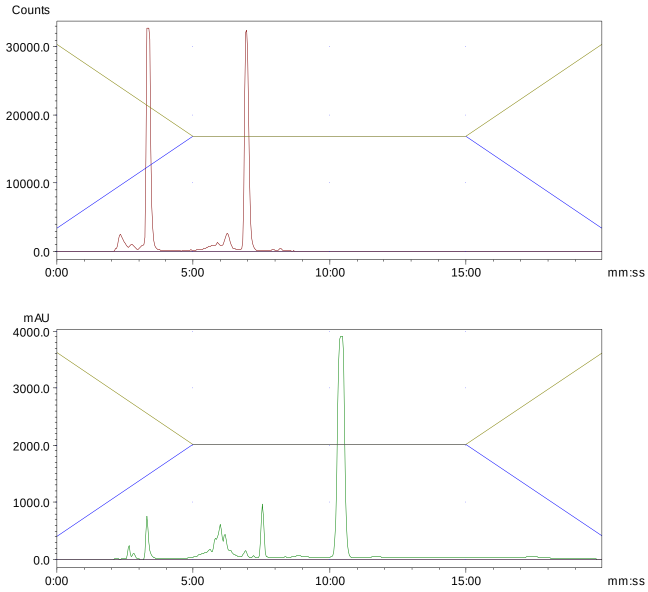

Figure S5: Representative analytical HPLC chromatogram for the attempted fluorination of the tosyl precursor **5.** a) Radiochromatogram showing unreacted [^{18}F]fluoride and formation of an unidentified radiolabelled by-product b) UV chromatogram showing mostly unreacted tosyl precursor.

Precursor	Solvent	Mg	T(°C)	Time	$(^{18}F^-)$	^{18}F-FLTProtide	^{18}F-by-products
6	CH_3CN	10 mg	90°C	15min	1.2 GBq	No	Yes
6	CH_3CN	10 mg	90°C	20min	1.5 GBq	Yes	Yes
6	CH_3CN	10 mg	90°C	30min	2.3 GBq	Yes	Yes
6	CH_3CN	10 mg	90°C	40min	2.2 GBq	Yes	Yes
6	DMF	10 mg	120°C	15min	734 MBq	No	Yes
6	DMF	10 mg	120°C	20min	1.1 GBq	No	Yes

Table S3: Radiolabeling attempts for the unprotected nosyl precursor (compound **6**).

a)

b)

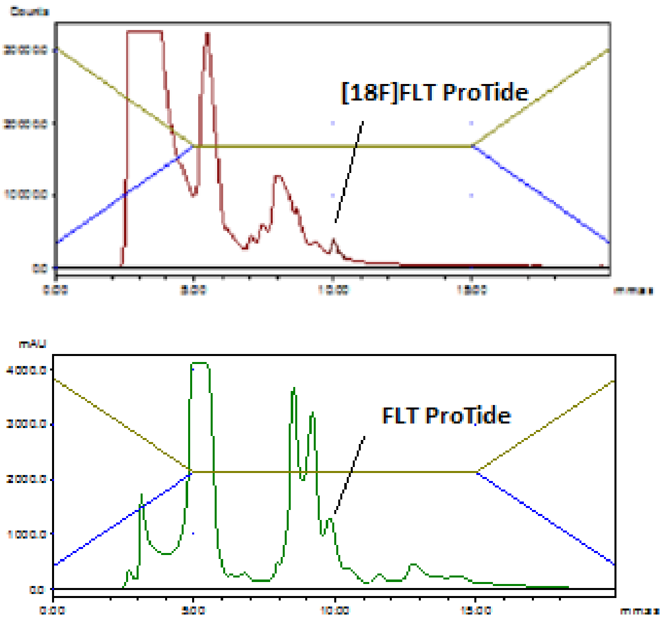

Figure S6: Representative analytical HPLC chromatogram for the attempted fluorination of the nosyl unprotected precursor **6.** a) Radiochromatogram showing unreacted [^{18}F]fluoride, formation of several radiolabelled by-products and formation of <1% radiolabelled product. b) UV chromatogram of the reaction mixture co-spiked with the non-radioactive standard to identify product FLT ProTide.

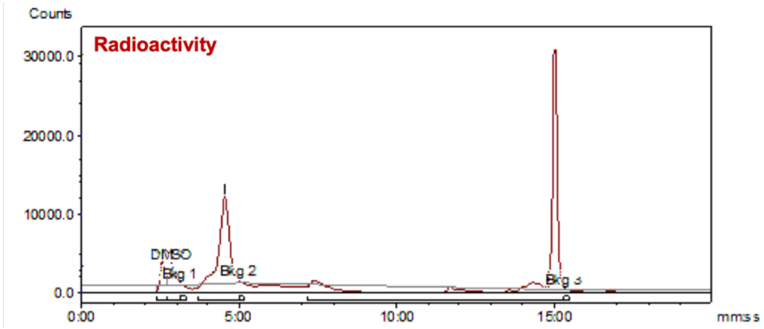

Figure S7: Representative analytical HPLC chromatogram for the fluorination of the nosyl protected precursor **7.** Radiochromatogram showing formation of the radiolabelled protected product (R_t = 15min).

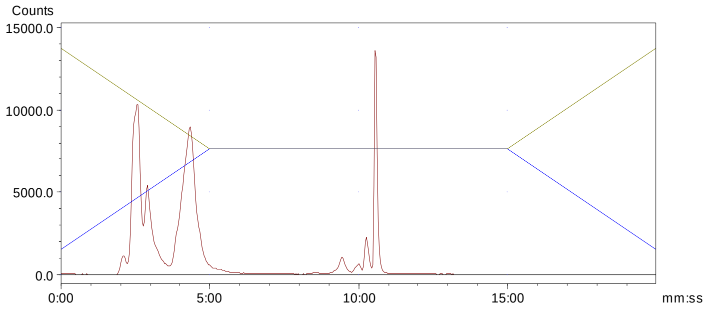

Figure S8: Representative analytical HPLC chromatogram for the deprotection of the precursor **15** before purification**.** The radiochromatogram shows formation of desired radiolabelled [^{18}F]FLT ProTide product **1**.

a) b)

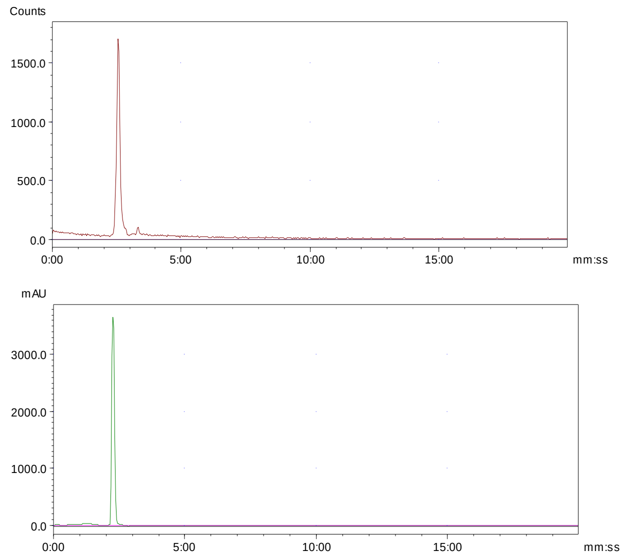

Figure S9: Representative analytical HPLC chromatogram for the fluorination of the sugar**.** a) The radiochromatogram shows fully converted product. b) UV chromatogram of the reaction mixture co-spiked with the commercially available cold standard.

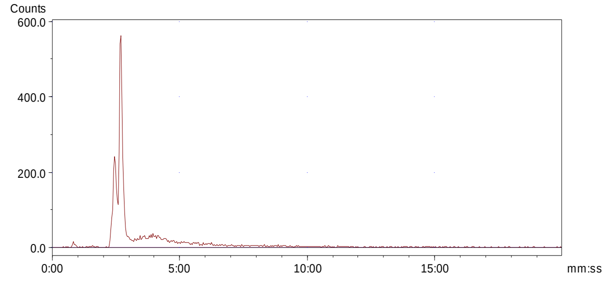

Figure S10: Representative analytical HPLC chromatogram of the glycosylation reaction**.** The radiochromatogram shows formation of two anomers of which the major (**24**) is the β.

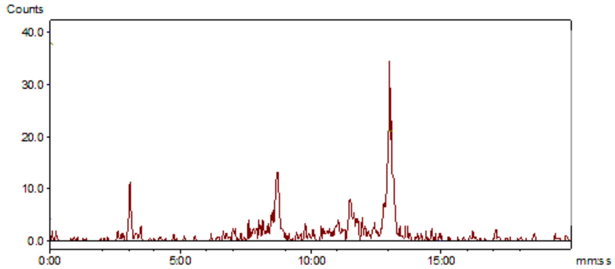

Figure S11: Representative analytical HPLC chromatogram of the coupling reaction**.** The radiochromatogram of the crude mixture shows formation of compound **2**.
